# Differential methylation analysis of reduced representation bisulfite sequencing experiments using edgeR

**DOI:** 10.12688/f1000research.13196.2

**Published:** 2018-10-08

**Authors:** Yunshun Chen, Bhupinder Pal, Jane E. Visvader, Gordon K. Smyth

**Affiliations:** 1 The Walter and Eliza Hall Institute of Medical Research, Parkville, VIC, 3052, Australia; 2 Department of Medical Biology, The University of Melbourne, Melbourne, VIC, 3010, Australia; 3 School of Mathematics and Statistics, The University of Melbourne, Melbourne, VIC, 3010, Australia

**Keywords:** Methylation, BS-seq, differential methylation analysis, Bioconductor

## Abstract

Cytosine methylation is an important DNA epigenetic modification. In vertebrates, methylation occurs at CpG sites, which are dinucleotides where a cytosine is immediately followed by a guanine in the DNA sequence from 5' to 3'. When located in the promoter region of a gene, DNA methylation is often associated with transcriptional silencing of the gene. Aberrant DNA methylation is associated with the development of various diseases such as cancer. Bisulfite sequencing (BS-seq) is the current "gold-standard" technology for high-resolution profiling of DNA methylation. Reduced representation bisulfite sequencing (RRBS) is an efficient form of BS-seq that targets CpG-rich DNA regions in order to save sequencing costs. A typical bioinformatics aim is to identify CpGs that are differentially methylated (DM) between experimental conditions. This workflow demonstrates that differential methylation analysis of RRBS data can be conducted using software and methodology originally developed for RNA-seq data. The RNA-seq pipeline is adapted to methylation by adding extra columns to the design matrix to account for read coverage at each CpG, after which the RRBS and RNA-seq pipelines are almost identical. This approach is statistically natural and gives analysts access to a rich collection of analysis tools including generalized linear models, gene set testing and pathway analysis. The article presents a complete start to finish case study analysis of RRBS profiles of different cell populations from the mouse mammary gland using the Bioconductor package edgeR. We show that lineage-committed cells are typically hyper-methylated compared to progenitor cells and this is true on all the autosomes but not the sex chromosomes. We demonstrate a strong negative correlation between methylation of promoter regions and gene expression as measured by RNA-seq for the same cell types, showing that methylation is a regulatory mechanism involved in epithelial linear commitment.

## Introduction

Cytosine methylation is an important epigenetic DNA modification that is generally associated with transcriptional silencing ^1^. In vertebrates, methylation occurs at CpG sites, which are dinucleotides where a cytosine (C) is immediately followed by a guanine (G) in the DNA sequence from 5’ to 3’. CpG dinucleotides are relatively uncommon on the human genome but occur more frequently in gene promoters and exons ^2^. About 72% of human gene promoters are enriched for CpGs ^2^. CpGs in gene promoters tend to cluster in CpG islands (CGIs), which are regions of a few hundred to a couple of thousand base pairs with very strong enrichment of CpGs ^3^.

The relationship of DNA methylation to transcription in vertebrates is complex ^4^. Methylation of CGIs causes robust transcriptional repression and is required for long-term mono-allelic silencing including X inactivation and genomic imprinting ^1^. Methylation of CpG-poor promoters is more weakly associated with gene expression, and the mechanisms by which this occurs are unclear ^1^. Methylation of gene bodies on the other hand can be positively associated with gene expression ^4, 5^.

DNA methylation is relatively stable in that most CGIs do not change methylation state during normal cell development. Nevertheless DNA methylation is understood to play a regulatory role in differentiation and commitment in adult cell lineages ^6, 7^. Aberrant methylation patterns are also associated with the development of diseases such as cancer ^8, 9^.

Bisulfite sequencing (BS-seq) is increasingly used to profile DNA methylation ^10, 11^. Unmethylated cytosines (C) are converted to Uracils (U) by sodium bisulfite and then deaminated to thymines (T) during PCR amplification. Methylated Cs, on the other hand, remain intact after bisulfite treatment. This strategy produces whole genome bisulfite sequencing (WGBS) when combined with sequencing of the entire genome. WGBS is sometimes considered the “gold standard” for methylation profiling because it provides single-nucleotide resolution and whole-genome coverage ^11^. However it requires large quantities of DNA and is expensive because of the amount of sequencing required.

The fact that CpG islands constitute only a small percentage of the genome makes the WGBS approach inefficient in terms of information content per sequenced read. To improve efficiency and reduce costs, enrichment strategies have been developed and combined with BS-seq to target a specific fraction of the genome. A common targeted approach is reduced representation bisulfite sequencing (RRBS) that targets CpG-rich regions ^11, 12^. Under the RRBS strategy, small fragments that compose only 1% of the genome are generated using MspI digestion, which means fewer reads need to be sequenced in total to provide reasonable coverage of the targeted regions. The RRBS approach can capture approximately 70% of gene promoters and 85% of CpG islands, while requiring only small quantities of input sample ^13^. RRBS has great advantages in cost and efficiency, especially when CGI or gene-orientated results are required. RRBS is also applicable for single cell studies ^14^.

The first step of analyzing BS-seq data is to align short sequence reads to a reference genome. The number of C-to-T conversions are then counted for all the mapped reads. A number of software tools have been developed to facilitate read mapping and methylation calling, including *Bismark* ^15^, *MethylCoder* ^16^, *BRAT* ^17^, *BSSeeker* ^18, 19^ and *BSMAP* ^20^. Most of these tools rely on existing short read aligners, such as Bowtie ^21, 22^.

Typical downstream DNA methylation studies often involve finding differentially methylated regions (DMRs) between different experimental conditions. A number of statistical methods and software packages have been developed for detecting DMRs using the BS-seq technology. *methylkit* ^23^ and *RnBeads* ^24^ implement Fisher’s Exact Test, which is a popular choice for two-group comparisons with no replicates. In the case of complex experimental designs, regression methods are widely used to model methylation levels or read counts. *RnBeads* offers a linear regression approach based on the moderated t-test and empirical Bayes method implemented in *limma* ^25^. *BSmooth* ^26^ is another analysis pipeline that uses linear regression together with a local likelihood smoother. *methylkit* also has an option to apply logistic regression with overdispersion correction ^23^. Some other methods have been developed based on beta-binomial distribution to achieve better variance modeling. For example, *DSS* fits a Bayesian hierarchical beta-binomial model to BS-seq data and uses Wald tests to detect DMRs ^27^. Other software using beta-binomial model include *BiSeq* ^28^, *MOABS* ^29^ and *RADMeth* ^30^.

In this workflow, we demonstrate an *edgeR* pipeline for differential methylation analysis. *edgeR* is one of the most popular Bioconductor packages for assessing differential expression in RNA-seq data ^31, 32^. It is based on the negative binomial (NB) distribution and it models the variation between biological replicates through the NB dispersion parameter. Unlike other approaches to methylation sequencing data, the analysis explained in this workflow keeps the counts for methylated and unmethylated reads as separate observations. *edgeR* linear models are used to fit the total read count (methylated plus unmethylated) at each genomic locus, in such a way that the proportion of methylated reads at each locus is modeled indirectly as an over-dispersed binomial-like distribution. This approach has a number of advantages. First, it allows the differential methylation analysis to be undertaken using existing *edgeR* pipelines developed originally for RNA-seq differential expression analyses. The *edgeR* generalized linear model (GLM) framework offers great flexibility for analysing complex experimental designs while still accounting for the biological variability ^33^. Second, keeping methylated and unmethylated read count as separate data observations allows the inherent variability of the data to be modeled more directly and perhaps more realistically. Differential methylation is assessed by likelihood ratio tests so we do not need to assume that the log-fold-changes or other coefficient estimators are normally distributed.

This article presents an analysis of an RRBS data set generated by the authors containing replicated RRBS profiles of basal and luminal cell populations from the mouse mammary epithelium. Our primary interest is in gene-orientated and pathway-orientated interpretations of the result. It is of particular importance to relate methylation changes to RNA-seq expression changes for the same genes. We show how to analyze differential methylation changes either for individual CpGs or for pre-specified genomic regions, such as chromosomes or genes, and we particularly focus on methylation changes by promoter regions.

As with other articles in the Bioconductor Gateway series, our aim is to provide an example analysis with complete start to finish code. As with other Bioconductor workflow articles, we illustrate one analysis strategy in detail rather than comparing different pipelines. The analysis approach illustrated in this article can in principle be applied to any BS-seq data but is especially appropriate for RRBS data. The approach is designed for experiments that included biological replication but can be used without replication if the NB dispersion is preset. The results shown in this article were generated using Bioconductor Release 3.7.

The next section gives an expository introduction to the *edgeR* approach to methylation data. The analysis of the mammary epithelial data starts afterwards.

## Introducing the NB linear modeling approach to BS-seq data

### A very small example

To introduce the *edgeR* linear modeling approach to BS-seq data, consider a genomic locus that has *mA* methylated and *u _A_* unmethylated reads in condition A and *m _B_* methylated and *u _B_* unmethylated reads in condition B. Our approach is to model all four counts as NB distributed with the same dispersion but different means. Suppose the data is as given in Table 1. The counts can be entered into a matrix in R:

> counts <- matrix(c(2,12,11,0),1,4)
> dimnames(counts) <- list("Locus", c("A.Me","A.Un","B.Me","B.Un"))
> counts

       A.Me A.Un B.Me B.Un
Locus    2   12   11    0

**Table 1.** A very small example data set.

Sample	Condition	Methylated Count	Unmethylated Count
1	A	2	12
2	B	11	0

The experimental design of the data can be summarized by indicating which counts correspond to each sample and each condition:

> design <- cbind(Sample1 = c(1,1,0,0),
+                   Sample2 = c(0,0,1,1),
+                   A = c(1,0,0,0),
+                   B = c(0,0,1,0))

In the design matrix, the first two columns are indicators for samples 1 and 2 while the third and fourth columns indicate the methylated count columns for conditions A and B.

If this were a complete data set, then it could be analyzed in *edgeR* as follows. First we fit a negative binomial generalized linear model to the counts:

> library(edgeR)
> fit <- glmFit(counts, design, lib.size=c(100,100,100,100), dispersion=0.0247)

The first two coefficients in the linear model are used to model the total number of reads (methylated or unmethylated) for samples 1 and 2, respectively. Coefficient 3 estimates the log ratio of methylated to unmethylated reads for condition A, a quantity that can also be viewed as the logit proportion of methylated reads in condition A. In mathematical terms, we have ${\overset{\wedge }{\beta }}_{A}={\text{log}}_{2}(2.125/12.125)=-2.51$. (Note that *edgeR* adds 0.125 to each count when reporting the coefficients to avoid taking logarithms of zero.) Coefficient 4 estimates the log ratio of methylated to unmethylated reads for condition B, in mathematical terms ${\overset{\wedge }{\beta }}_{B}={\text{log}}_{2}(11.125/0.125)=6.48$.

Then we conduct a likelihood ratio test to compare the methylation rate in condition B to that in condition A:

> lrt <- glmLRT(fit, contrast = c(0,0,-1,1))
> topTags(lrt)

Coefficient:  -1*A 1*B
      logFC logCPM   LR   PValue      FDR
Locus  8.99   16.3 20.7 5.27e-06 5.27e-06

The contrast vector indicates that we wish to compare the fourth to the third coefficient, ignoring the two sample coverage coefficients. The estimated value for the contrast is the difference in logit methylation rates, which is estimated as ${\overset{\wedge }{\beta }}_{B}-{\overset{\wedge }{\beta }}_{A}=6.48-(\text{− 2.51})=8.99$, a quantity that is shown as the log2-fold-change (logFC) column of the results table. The chisquare likelihood ratio statistic (LR) is 20.7 and the P-value for differential methylation in condition B vs condition A is *P* = 5.27 *×* 10 ^*−*6^.

Note that the specific parametrization used to account for the sample effects is not important. The test for differential methylation would be unchanged if we represented the two sample effects using an intercept model. Indeed, the first two columns of the design matrix could be any two columns spanning the sample effects:

> design <- cbind(Intercept = c(1,1,1,1),
+                   Sample2 = c(0,0,1,1),
+                   A = c(1,0,0,0),
+                   B = c(0,0,1,0))
> fit <- glmFit(counts, design, lib.size=c(100,100,100,100), dispersion=0.0247)
> lrt <- glmLRT(fit, contrast = c(0,0,-1,1))
> topTags(lrt)

Coefficient:  -1*A 1*B
      logFC logCPM   LR   PValue      FDR
Locus  8.99   16.3 20.7 5.27e-06 5.27e-06

The dispersion parameter controls the degree of biological variability ^33^. If we had set dispersion=0 in the above code, then the above analysis would be exactly equivalent to a logistic binomial regression, with the methylated counts as responses and the total counts as sizes, and with a likelihood ratio test for a difference in proportions between conditions A and B. Positive values for the dispersion produce over-dispersion relative to the binomial distribution. We have set the dispersion here equal to the value that is estimated below for the mammary epithelial data.

In the above code, the two library sizes for each sample should be equal. Otherwise, the library size values are arbitrary and any settings would lead to the same P-value.

### Relationship to beta-binomial modeling

It is interesting to compare this approach with beta-binomial modeling. It is well known that if *m* and *u* are independent Poisson random variables with means *µ _m_* and *µ _u_*, then the conditional distribution of *m* given *m* + *u* is binomial with success probability *p* = *µ _m_/*( *µ _m_* + *µ _u_*). If the Poisson means *µ _m_* and *µ _u_* themselves follow gamma distributions, then the marginal distributions of *m* and *u* are NB instead of Poisson. If the two NB distributions have different dispersions, and have expected values in inverse proportion to the dispersions, then the conditional distribution of *m* given *m* + *u* follows a beta-binomial distribution. The approach taken in this article is closely related to the beta-binomial approach but makes different and seemingly more natural assumptions about the NB distributions. We instead assume the two NB distributions to have the same dispersion but different means. The NB linear modeling approach allows the means and dispersions of the two NB distributions to be estimated separately, in concordance with the data instead of being artificially linked.

### A small example with replicates

*edgeR* linear modeling takes advantage of replicate libraries for each condition. Now we augment the small example above to include two replicates for each experimental condition. There are now four samples and eight counts ( Table 2). The eight counts can be represented in R as follows:

> counts <- matrix(c(2,12,4,20,11,0,15,3),1,8)
> row.names(counts) <- "Locus"
> colnames(counts) <- c("A1.Me","A1.Un","A2.Me","A2.Un",
+                          "B1.Me","B1.Un","B2.Me","B2.Un")
> counts

      A1.Me A1.Un A2.Me A2.Un B1.Me B1.Un B2.Me B2.Un
Locus     2    12     4    20    11     0    15     3

**Table 2.** A slightly less small example data set with *n* = 2 replicates for each condition.

Sample	Condition	Methylated Count	Unmethylated Count
1	A	2	12
2	A	4	20
3	B	11	0
4	B	15	3

We can represent the sample information associated with the counts as follows:

> Sample <- gl(4,2,8)
> Methylation <- gl(2,1,8, labels=c("Me","Un"))
> Condition <- gl(2,4,8, labels=c("A","B"))
> SampleInfo <- data.frame(Sample,Methylation,Condition)
> row.names(SampleInfo) <- colnames(counts)
> SampleInfo

       Sample Methylation Condition
A1.Me      1          Me         A
A1.Un      1          Un         A
A2.Me      2          Me         A
A2.Un      2          Un         A
B1.Me      3          Me         B
B1.Un      3          Un         B
B2.Me      4          Me         B
B2.Un      4          Un         B

The design matrix is a simple combination of two parts. First there is a matrix to model the sample coverages:

> design.samples <- model.matrix(~0+Sample)

Then there is a matrix to model the two treatment conditions.

> design.conditions <- model.matrix(~0+Condition)

The full matrix is constructed from the two parts, with the condition design matrix mediating the methylation effects:

> design <- cbind(design.samples, (Methylation=="Me") * design.conditions)
> design

   Sample1 Sample2 Sample3 Sample4 ConditionA ConditionB
1        1       0       0       0          1          0
2        1       0       0       0          0          0
3        0       1       0       0          1          0
4        0       1       0       0          0          0
5        0       0       1       0          0          1
6        0       0       1       0          0          0
7        0       0       0       1          0          1
8        0       0       0       1          0          0

Now we can fit the GLM and conduct the statistical test for differential methylation:

> fit <- glmFit(counts, design, lib.size=100, dispersion=0.0247)
> contBvsA <- makeContrasts(BvsA = ConditionB - ConditionA, levels=design)
> lrt <- glmLRT(fit, contrast=contBvsA)
> topTags(lrt)

Coefficient:  -1*ConditionA 1*ConditionB
      logFC logCPM   LR   PValue      FDR
Locus   5.4   16.6 34.3 4.84e-09 4.84e-09

The results for each condition have been averaged over the replicates. The change in methylation is now of smaller magnitude than for Table 1, as shown by the smaller logFC value, but the P-value is more significant because of the greater confidence that comes from the extra replicates.

### Automatic construction of the design matrix

In this expository introduction we have demonstrated the construction of the design matrix from basic principles. In practice, *edgeR* provides a function modelMatrixMeth to automate the construction of the design matrix. The user can simply specify the treatment conditions associated with each DNA sample (as in the second column of Table 2):

> Condition <- factor(c("A","A","B","B"))

Then the design matrix is constructed by:

> design <- modelMatrixMeth(~0+Condition)
> design

  Sample1 Sample2 Sample3 Sample4 ConditionA ConditionB
1       1       0       0       0          1          0
2       1       0       0       0          0          0
3       0       1       0       0          1          0
4       0       1       0       0          0          0
5       0       0       1       0          0          1
6       0       0       1       0          0          0
7       0       0       0       1          0          1
8       0       0       0       1          0          0

Alternatively, a user can construct any appropriate design matrix at the sample level (with 4 rows), exactly as one would do for a RNA-seq differential expression analysis, then the function will expand the sample-level design matrix to the appropriate observation-level matrix with 8 rows:

> designSL <- model.matrix(~0+Condition)
> design <- modelMatrixMeth(designSL)

This gives exactly the same result as above.

### Another way to make the design matrix

When there are just two treatment conditions to be compared, it can be slightly simpler to compute the log2-fold-change directly as a coefficient of the linear model, rather than forming it as a contrast. This can be done by including an intercept in the design matrix as follows:

> design <- modelMatrixMeth(~Condition)
> design

  Sample1 Sample2 Sample3 Sample4 (Intercept) ConditionB
1       1       0       0       0           1          0
2       1       0       0       0           0          0
3       0       1       0       0           1          0
4       0       1       0       0           0          0
5       0       0       1       0           1          1
6       0       0       1       0           0          0
7       0       0       0       1           1          1
8       0       0       0       1           0          0

In this formulation, the second last coefficient still estimates the logit methylation level in condition A, but the last coefficient now estimates the log2-fold-change directly:

> fit <- glmFit(counts, design, lib.size=100, dispersion=0.0247)
> lrt <- glmLRT(fit, coef="ConditionB")
> topTags(lrt)

Coefficient:  ConditionB
      logFC logCPM   LR   PValue      FDR
Locus   5.4   16.6 34.3 4.84e-09 4.84e-09

Again, this gives the same results as the previous analysis.

### Analogy with paired-samples expression analyses

The above design matrix might seem as if it is very special to methylation data, but in fact the same sort of design matrix would be used for any gene expression analysis when there are paired-samples and we wish to compare treatment effects between groups. To see this, consider the small RNA-seq experiment shown in Table 3. This experiment has four subjects, each of whom contributes both treated and untreated RNA samples. The subjects belong to two groups (A and B) and we wish to test whether the treatment effect differs between the groups.

**Table 3.** A hypothetical paired-samples RNA-seq experiment.

Subject	Group	Treated	Untreated
1	A	2	12
2	A	4	20
3	B	11	0
4	B	15	3

Readers will note that Table 3 is the same as Table 2 except that the columns have been relabeled. Here, the four subjects are analogous to the four DNA samples in Table 2, the groups are analogous to conditions, and treatment is analogous to methylation. Exactly the same design matrix, as used above for the methylation experiment, would be appropriate here for the expression experiment. In *edgeR*, the only difference between the methylation and expression analyses would be in the normalization steps. The linear modeling and testing steps would be identical.

This shows that BS-seq data has a structure that is already familiar from RNA-seq experiments with paired-samples. The theme of this article is that statistical analysis methods developed for RNA-seq can be beneficially applied to BS-seq data.

### NB GLMs

In practice, the data will consist of many thousands of genomic loci and the counts matrix will have a row for each locus. The function glmFit fits a NB GLM to each row of read counts. If *y _gi_* is the *i*th read count for genomic locus *g*, then *y _gi_* is assumed to be NB distributed with expected value *µ _gi_* and dispersion *φ _g_*. The expected values are modeled by the design matrix,

$$\log\mu_{gi}=\sum_{j=1}^{p}x_{ij}\beta_{gj}+\log N_{i}$$

where the *x _ij_* are the *i*th row of the design matrix, *β _gj_* are the regression coefficients and *N _i_* are the library sizes. In the small example above with four samples, the design matrix had 6 columns, so *p* = 6. In general, if we have an experiment comparing *k* treatment conditions using *n* samples, then the count matrix will have 2 *n* columns and the design matrix will have *p* = *n* + *k* columns. The first *n* coefficients capture sample effects in read abundance, while the remaining *k* coefficients compare the methylation proportions between the conditions.

### Dispersion estimation

In the small example above we did not estimate the dispersion but simply used a preset value. In practice, there will not only be replicates for each genomic locus but also many thousands of loci. The *edgeR* package estimates a dispersion for each locus from the replicate variability, using an empirical Bayes procedure to pool information across all the loci while estimating locus-specific dispersion estimates ^33– 35^. The full data example on mouse mammary epithelial cells will illustrate this.

The variance of the read counts is assumed to follow a quadratic mean-variance relationship

$$\mathrm{var}(y_{gi})=\mu_{gi}+\phi_{g}\mu_{gi}^{2}$$

where *ϕ _g_* is the dispersion for locus *g*. The first term follows from sequencing variability and the second from biological variability ^33^. The NB variance function captures the fact that larger counts have larger variances. The quadratic relationship captures the technical factors that affect variability, so that *ϕ _g_* itself reflects only the biological characteristics of each locus.

## RRBS profiling of mammary epithelial cells

### Aim of the study

We now describe the main data set to be analyzed in this article. The epithelium of the mammary gland exists in a highly dynamic state, undergoing dramatic morphogenetic changes during puberty, pregnancy, lactation and regression ^36^. Characterization of the lineage hierarchy of cells in the mammary epithelium is an important step toward understanding which cells are predisposed to oncogenesis. In this study, we profiled the methylation status of the two major functionally distinct epithelial compartments: basal and luminal cells. The basal cells were further divided into those showing high or low expression of the surface marker Itga5 as part of our investigation of heterogeneity within the basal compartment. We carried out global RRBS DNA methylation assays on two biological replicates of each of the three cell populations to determine whether the epigenetic machinery played a potential role in (i) differentiation of luminal cells from basal and (ii) any compartmentalization of the basal cells associated with Itga5. Of particular interest is to relate transcriptional changes to methylation changes for the same genes.

### Sample preparation

Inguinal mammary glands (minus lymph node) were harvested from FVB/N mice. All animal experiments were conducted using mice bred at and maintained in our animal facility according to the Walter and Eliza Hall Institute of Medical Research Animal Ethics Committee guidelines. Epithelial cells were suspended and fluorescence-activated cell sorting (FACS) was used to isolate basal and luminal cell populations ^37^. Genomic DNA (gDNA) was extracted from freshly sorted cells using the Qiagen DNeasy kit. Around 25ng gDNA input was subjected to DNA methylation analysis by BS-seq using the Ovation RRBS Methyl-seq kit from NuGEN. The process includes MspI digestion of gDNA, sequencing adapter ligation, end repair, bisulfite conversion, and PCR amplification to produce the final sequencing library. The Qiagen EpiTect Bisulfite kit was used for bisulfite-mediated conversion of unmethylated cytosines.

### Experimental design

There are three groups of samples: luminal population, Itga5- basal population and Itga5+ basal population. Two biological replicates were collected for each group. This experimental design is summarized in the table below.

> targets <- read.delim("targets.txt", row.names="Sample", stringsAsFactors=FALSE)
> targets

     Population     Description
P6_1         P6         Luminal
P6_4         P6         Luminal
P7_2         P7 Basal_Itga5_neg
P7_5         P7 Basal_Itga5_neg
P8_3         P8 Basal_Itga5_pos
P8_6         P8 Basal_Itga5_pos

The experiment has a simple one-way layout with three groups.

The sequencing was carried out on the Illumina NextSeq 500 platform. About 30 million 75bp paired-end reads were generated for each sample.

## Reading and annotating the methylation counts

### Processing the BS-seq FASTQ files with Bismark

The first step of the analysis is to map the sequence reads from the FASTQ files to the mouse genome and to perform methylation calls. This is the only step of the analysis that cannot currently be done in R.

Though many options are available, we used *Bismark* software ( https://www.bioinformatics.babraham.ac.uk/projects/bismark) to count the methylated and unmethylated reads at each genomic locus. *Bismark* was run using recommended default settings. We first ran trim_galore ( https://www.bioinformatics.babraham.ac.uk/projects/trim_galore/) to remove adapters and to trim poor quality reads. Then *Bismark* version v0.13.0 was used to align the reads to the mouse mm10 genome using Bowtie2 ^22^. Finally, methylation calls were made using bismark_methylation_extractor.

The *Bismark* output consists of one coverage file for each sample. Readers wishing to reproduce the analysis presented in this article can download the coverage files produced by *Bismark* from http://bioinf.wehi.edu.au/edgeR/F1000Research2017.

### Reading in the data

The *Bismark* coverage files are just tab-delimited text files and so can be read into a dataframe in R using read.delim. Each of the files has the following format:

> P6_1 <- read.delim("P6_1.bismark.cov.gz", header=FALSE, nrows=6)
> P6_1

     V1      V2      V3    V4 V5 V6
1 chr15 3051454 3051454  87.5 14  2
2 chr15 3051455 3051455 100.0  1  0
3 chr15 3051486 3051486 100.0 16  0
4 chr15 3051555 3051555  81.2 13  3
5 chr15 3051556 3051556 100.0  1  0
6 chr15 3051559 3051559  87.5 14  2

The columns in the coverage file represent: V1: chromosome number; V2: start position of the CpG site; V3: end position of the CpG site; V4: methylation proportion; V5: number of methylated Cs; V6: number of unmethylated Cs.

In practice, we only need to read in columns V1, V2, V5 and V6. Column V3 is the same as V2, and the methylation proportion V4 is just a function of V5 and V6.

We now read in the Bismark coverage files for all the samples. The *edgeR* function readBismark2DGE reads all the files and collates the counts for all the sample into one data object:

> Sample <- row.names(targets)
> files <- paste0(Sample,".bismark.cov.gz")
> yall <- readBismark2DGE(files, sample.names=Sample)

Reading P6_1.bismark.cov.gz
Reading P6_4.bismark.cov.gz
Reading P7_2.bismark.cov.gz
Reading P7_5.bismark.cov.gz
Reading P8_3.bismark.cov.gz
Reading P8_6.bismark.cov.gz
Hashing ...
Collating counts ...

> dim(yall)

[1] 3538117      12

The *edgeR* package stores the counts and associated annotation in a simple list-based data object called a “DGEList”. The count matrix is

> head(yall$counts)

              P6_1-Me P6_1-Un P6_4-Me P6_4-Un P7_2-Me P7_2-Un P7_5-Me P7_5-Un
chr15-3051454      14       2      18       0       6       0      15       1
chr15-3051455       1       0       1       0       0       0       0       0
chr15-3051486      16       0      17       1       6       0      16       0
chr15-3051555      13       3      18       0       5       1      16       0
chr15-3051556       1       0       1       0       0       0       0       0
chr15-3051559      14       2      15       3       5       1      15       1
              P8_3-Me P8_3-Un P8_6-Me P8_6-Un
chr15-3051454       8       0       3       0
chr15-3051455       0       0       1       0
chr15-3051486       8       0       3       0
chr15-3051555       8       0       3       0
chr15-3051556       0       0       1       0
chr15-3051559       8       0       3       0

There is a row for each CpG locus found in any of the files. There columns of methylated and unmethylated counts for each sample. The chromosomes and genomic loci are stored in the annotation data.frame:

> head(yall$genes)

                Chr   Locus
chr15-3051454 chr15 3051454
chr15-3051455 chr15 3051455
chr15-3051486 chr15 3051486
chr15-3051555 chr15 3051555
chr15-3051556 chr15 3051556
chr15-3051559 chr15 3051559

Summary information on each sample is stored in yall$samples:

> yall$samples$group <- factor(targets$Population)
> head(yall$samples)

        group lib.size norm.factors
P6_1-Me    P6 27803470            1
P6_1-Un    P6 33444764            1
P6_4-Me    P7 37579181            1
P6_4-Un    P7 35595908            1
P7_2-Me    P8 17902366            1
P7_2-Un    P8 15609315            1

### Filtering unassembled chromosomes

It is convenient to remove genomic segments that have not been assembled into any of the recognized chromosomes:

> keep <- rep(TRUE, nrow(yall))
> Chr <- as.character(yall$genes$Chr)
> keep[ grep("random",Chr) ] <- FALSE
> keep[ grep("chrUn",Chr) ] <- FALSE

For this analysis, we also remove the Y chromosome and mitochondrial DNA:

> keep[Chr=="chrY"] <- FALSE
> keep[Chr=="chrM"] <- FALSE
> table(keep)

keep
  FALSE    TRUE
   6285 3531832

Then we can subset the DGEList data object to remove the rows corresponding to the unwanted chromosomes:

> yall <- yall[keep,, keep.lib.sizes=FALSE]

The option keep.lib.sizes=FALSE causes the library sizes to be recomputed.

### Sort into genomic order

Just for convenience, we sort the DGEList so that all loci are in genomic order, from chromosome 1 to chromosome X:

> ChrNames <- paste0("chr",c(1:19,"X"))
> ChrNames

 [1] "chr1"  "chr2"  "chr3"  "chr4"  "chr5"  "chr6"  "chr7"  "chr8"  "chr9"
[10] "chr10" "chr11" "chr12" "chr13" "chr14" "chr15" "chr16" "chr17" "chr18"
[19] "chr19" "chrX"

> yall$genes$Chr <- factor(yall$genes$Chr, levels=ChrNames)
> o <- order(yall$genes$Chr, yall$genes$Locus)
> yall <- yall[o,]

### Gene annotation

We now annotate the CpG loci with the identity of the nearest gene. We search for the gene transcriptional start site (TSS) closest to each our CpGs:

> TSS <- nearestTSS(yall$genes$Chr, yall$genes$Locus, species="Mm")
> yall$genes$EntrezID <- TSS$gene_id
> yall$genes$Symbol <- TSS$symbol
> yall$genes$Strand <- TSS$strand
> yall$genes$Distance <- TSS$distance
> yall$genes$Width <- TSS$width
> head(yall$genes)

              Chr   Locus EntrezID Symbol Strand Distance  Width
chr1-3020689 chr1 3020689   497097   Xkr4      -  -650809 457017
chr1-3020690 chr1 3020690   497097   Xkr4      -  -650808 457017
chr1-3020708 chr1 3020708   497097   Xkr4      -  -650790 457017
chr1-3020724 chr1 3020724   497097   Xkr4      -  -650774 457017
chr1-3020725 chr1 3020725   497097   Xkr4      -  -650773 457017
chr1-3020814 chr1 3020814   497097   Xkr4      -  -650684 457017

Here EntrezID, Symbol, Strand and Width are the Entrez Gene ID, symbol, strand and width of the nearest gene. Distance is the genomic distance from the CpG to the TSS. Positive values means the TSS is downstream of the CpG and negative values means the TSS is upstream.

## Differential methylation analysis at CpG loci

### Filtering to remove low counts

We now turn to statistical analysis of differential methylation. Our first analysis will be for individual CpG loci.

CpG loci that have low coverage are removed prior to downstream analysis as they provide little information for assessing methylation levels. Filtering low-coverage CpGs also simplifies the subsequent analysis because of the reduction in data rows.

We sum up the counts of methylated and unmethylated reads to get the total read coverage at each CpG site for each sample:

> Coverage <- yall$counts[, Methylation=="Me"] + yall$counts[, Methylation=="Un"]
> head(Coverage)

             P6_1-Me P6_4-Me P7_2-Me P7_5-Me P8_3-Me P8_6-Me
chr1-3020689      16      21       4      13       6       2
chr1-3020690      37      47      18      46      15      22
chr1-3020708       0       2       0       1       0       0
chr1-3020724      16      21       4      14       6       2
chr1-3020725      37      47      18      46      15      22
chr1-3020814       0       0       0       1       0       0

The analysis needs to be restricted to CpG sites that have enough coverage for the methylation level to be measurable in a meaningful way at that site. As a conservative rule of thumb, we require a CpG site to have a total count (both methylated and unmethylated) of at least 8 in every sample before it is considered in the study.

> keep <- rowSums(Coverage >= 8) == 6
> table(keep)

keep
  FALSE    TRUE
3019830  512002

This filtering criterion could be relaxed somewhat in principle but the number of CpGs kept in the analysis is large enough for our purposes.

The DGEList object is subsetted to retain only the non-filtered loci:

> y <- yall[keep,, keep.lib.sizes=FALSE]

Again, the option keep.lib.sizes=FALSE causes the library sizes to be recomputed after the filtering. We generally recommend this, although the effect on the downstream analysis is usually small.

### Normalization

A key difference between BS-seq and other sequencing data is that the pair of libraries holding the methylated and unmethylated reads for a particular sample are treated as a unit. To ensure that the methylated and unmethylated reads for the same sample are treated on the same scale, we need to set the library sizes to be equal for each pair of libraries. We set the library sizes for each sample to be the average of the total read counts for the methylated and unmethylated libraries:

> TotalLibSize <- y$samples$lib.size[Methylation=="Me"] +
+                 y$samples$lib.size[Methylation=="Un"]
> y$samples$lib.size <- rep(TotalLibSize, each=2)
> y$samples
         
        group lib.size norm.factors
P6_1-Me    P6 27754786            1
P6_1-Un    P6 27754786            1
P6_4-Me    P7 41007143            1
P6_4-Un    P7 41007143            1
P7_2-Me    P8 21424225            1
P7_2-Un    P8 21424225            1
P7_5-Me    P6 26197143            1
P7_5-Un    P6 26197143            1
P8_3-Me    P7 18782105            1
P8_3-Un    P7 18782105            1
P8_6-Me    P8 15539660            1
P8_6-Un    P8 15539660            1

Other normalization methods developed for RNA-seq data, such as TMM ^38^, are not required for BS-seq data.

### Exploring differences between samples

In microarray methylation studies, a common measure of methylation level is the M-value, which is defined as *M* = log _2_ {(Me + *α*) */*(Un + *α*)} where Me and Un are the methylated and unmethylated intensities and *α* is some suitable offset to avoid taking logarithms of zero ^39^. The M-value can be interpreted as the base2 logit transformation of the proportion of methylated signal at each locus.

We compute the corresponding methylation summary from the methylated and unmethylated counts.

We compute the corresponding methylation summary from the methylated and unmethylated counts.

> Me <- y$counts[, Methylation=="Me"]
> Un <- y$counts[, Methylation=="Un"]
> M <- log2(Me + 2) - log2(Un + 2)
> colnames(M) <- Sample

Here M contains the empirical logit methylation level for each CpG site in each sample. We have used a prior count of 2 to avoid logarithms of zero. The exact value of the prior count is unimportant, but a value of 2 is common in other contexts such as RNA-seq.

**Figure 1.**
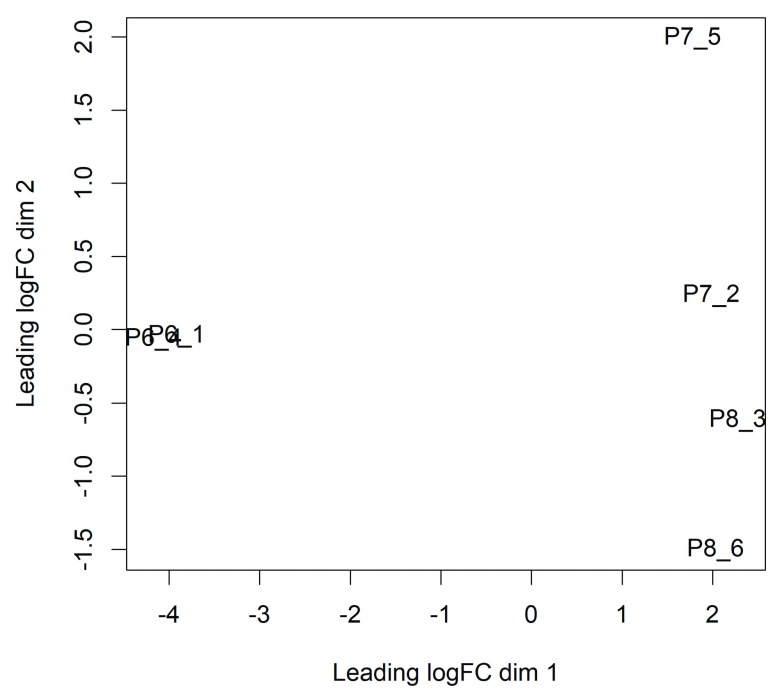
MDS plots showing overall differences in methylation levels between the samples. Replicate samples from the same population cluster together. The first dimension separates the luminal cells from the basal cells. The second dimension separates the Itga+ basal cells at the bottom from the Itga- basal cells at the top.

Now we can generate a multi-dimensional scaling (MDS) plot to explore the overall differences between the methylation levels of the different samples ( Figure 1):

> plotMDS(M)

In this plot the distance between each pair of samples represents the average logit change between the samples for the top most differentially methylated CpG loci between that pair of samples. (We call this average the *leading log-fold-change*.) The two replicate samples from the luminal population (P6) are seen to be well separated from the four basal samples (populations P7 and P8), with a leading change of about 6 logit units.

### Design matrix

One aim of this study is to identify differentially methylated (DM) loci between the different cell populations. In *edgeR*, this can be done by fitting linear models under a specified design matrix and testing for corresponding coefficients or contrasts. A basic sample-level design matrix can be made as follows:

> designSL <- model.matrix(~0+Population, data=targets)
> colnames(designSL) <- c("P6","P7","P8")
> designSL

     P6 P7 P8
P6_1  1  0  0
P6_4  1  0  0
P7_2  0  1  0
P7_5  0  1  0
P8_3  0  0  1
P8_6  0  0  1
attr(,"assign")
[1] 1 1 1
attr(,"contrasts")
attr(,"contrasts")$Population
[1] "contr.treatment"

The we expand this to the full design matrix modeling the sample and methylation effects:

> design <- modelMatrixMeth(designSL)
> design

   Sample1 Sample2 Sample3 Sample4 Sample5 Sample6 P6 P7 P8
1        1       0       0       0       0       0  1  0  0
2        1       0       0       0       0       0  0  0  0
3        0       1       0       0       0       0  1  0  0
4        0       1       0       0       0       0  0  0  0
5        0       0       1       0       0       0  0  1  0
6        0       0       1       0       0       0  0  0  0
7        0       0       0       1       0       0  0  1  0
8        0       0       0       1       0       0  0  0  0
9        0       0       0       0       1       0  0  0  1
10       0       0       0       0       1       0  0  0  0
11       0       0       0       0       0       1  0  0  1
12       0       0       0       0       0       1  0  0  0

The first six columns represent the sample coverage effects. The last three columns represent the methylation levels (in logit units) in the three cell populations.

### Dispersion estimation

With the design matrix specified, we can now proceed to the standard *edgeR* pipeline and analyze the data in the same way as for RNA-seq data. Similar to the RNA-seq data, the variability between biological replicates has also been observed in bisulfite sequencing data. This variability can be captured by the NB dispersion parameter under the generalized linear model (GLM) framework in *edgeR*.

The mean-dispersion relationship of BS-seq data has been studied in the past and no apparent mean-dispersion trend was observed ^27^. This is also verified through our own practice. Therefore, we would not consider a mean-dependent dispersion trend as we normally would for RNA-seq data. A common dispersion estimate for all the loci, as well as an empirical Bayes moderated dispersion for each individual locus, can be obtained from the estimateDisp function in *edgeR*:

> y <- estimateDisp(y, design, trend="none")
> y$common.dispersion

[1] 0.0275

> y$prior.df

[1] Inf

This returns a DGEList object with additional components ( common.dispersion and tagwise.dispersion) added to hold the estimated dispersions. Here the estimation of trended dispersion has been turned off by setting trend="none". For this data, the estimated prior degrees of freedom (df) are infinite for all the loci, which implies all the CpG-wise dispersions are equal to the common dispersion. A BCV plot is often useful to visualize the dispersion estimates, but is not informative in this case.

### Testing for differentially methylated CpG loci

We first fit NB GLMs for all the CpG loci using the glmFit function in *edgeR*.

> fit <- glmFit(y, design)

Then we can proceed to testing for differentially methylated CpG sites between different populations. One of the most interesting comparisons is between the basal (P7 and P8) and luminal (P6) populations. The contrast corresponding to any specified comparison can be constructed conveniently using the makeContrasts function:

> contr <- makeContrasts(LvsB=P6-0.5*(P7+P8), levels=design)

The actual testing is performed using likelihood ratio tests (LRT) in *edgeR*:

> lrt <- glmLRT(fit, contrast=contr)

The top set of most differentially methylated (DM) CpG sites can be viewed with topTags:

> topTags(lrt)

Coefficient:  1*P6 -0.5*P7 -0.5*P8
                  Chr     Locus EntrezID Symbol Strand Distance  Width logFC
chr16-76326604  chr16  76326604   268903  Nrip1      -   -46445  85647 -8.87
chr13-45709467  chr13  45709467    66355   Gmpr      +  -202023  38943  7.60
chr10-40387375  chr10  40387375    78334  Cdk19      +   -38067 134511 -8.13
chr11-100144651 chr11 100144651    16669  Krt19      -      952   2889 -8.19
chr17-46572098  chr17  46572098    20807    Srf      -    15936   9324  9.10
chr13-45709489  chr13  45709489    66355   Gmpr      +  -202045  38943  7.47
chr3-54724012    chr3  54724012    69639 Exosc8      -   -11352   6686 -8.40
chr13-45709480  chr13  45709480    66355   Gmpr      +  -202036  38943  7.70
chr8-120068504   chr8 120068504   102193 Zdhhc7      -   -32968  20378  7.42
chr2-69631013    chr2  69631013    72569   Bbs5      +    16242  20315 -7.30
                logCPM  LR   PValue      FDR
chr16-76326604    1.52 321 8.35e-72 4.27e-66
chr13-45709467    1.98 319 2.62e-71 6.70e-66
chr10-40387375    1.77 312 6.46e-70 1.10e-64
chr11-100144651   1.58 306 1.40e-68 1.79e-63
chr17-46572098    1.61 302 1.44e-67 1.48e-62
chr13-45709489    1.97 294 5.46e-66 4.66e-61
chr3-54724012     1.31 292 1.56e-65 1.14e-60
chr13-45709480    1.97 292 2.01e-65 1.28e-60
chr8-120068504    1.73 285 7.12e-64 4.05e-59
chr2-69631013     2.03 277 3.66e-62 1.88e-57

Here positive log-fold-changes represent CpG sites that have higher methylation level in the luminal population compared to the two basal populations. The Benjamini-Hochberg multiple testing correction is applied to control the false discovery rate (FDR).

The total number of DM CpG sites identified at an FDR of 5% can be shown with decideTests. There are in fact more than 50,000 differentially methylated CpGs in this comparison:

> summary(decideTests(lrt))

       1*P6 -0.5*P7 -0.5*P8
Down                  20302
NotSig               451589
Up                    40111

**Figure 2.**
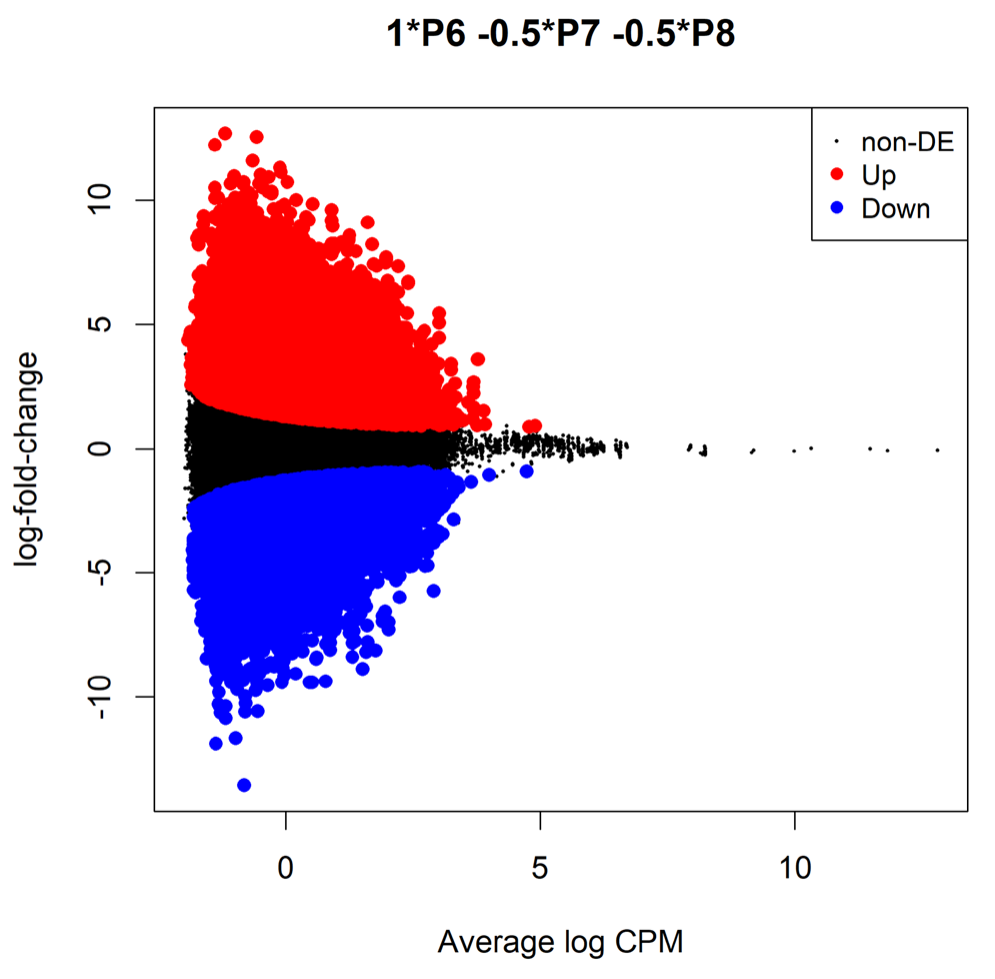
MD plot showing the log-fold-change of the methylation level and average abundance of each CpG site. Significantly hyper- and hypomethylated CpGs are highlighted in red and blue, respectively.

The differential methylation results can be visualized with an MD plot (see Figure 2):

> plotMD(lrt)

The logFC of the methylation level for each CpG site is plotted against the average abundance in log2-CPM. Significantly differentially methylated CpGs are highlighted.

### Differential methylation by chromosome

We now explore overall methylation patterns by chromosome. To do this we make a list of index vectors, with each vector identifying the loci that belong to that chromosome:

> ChrIndices <- list()
> for (a in ChrNames) ChrIndices[[a]] <- which(y$genes$Chr==a)

Then we can conduct “fry” gene set tests to evaluate overall changes in methylation in each chromosome:

> fry(y, index=ChrIndices, design=design, contrast=contr)

      NGenes Direction  PValue     FDR PValue.Mixed FDR.Mixed
chr18  16963        Up 0.00110 0.00543     1.02e-04  1.15e-04
chr15  22338        Up 0.00165 0.00543     8.53e-05  1.15e-04
chr10  27969        Up 0.00264 0.00543     1.03e-04  1.15e-04
chr7   31266        Up 0.00276 0.00543     1.08e-04  1.15e-04
chr2   36289        Up 0.00284 0.00543     7.08e-05  1.15e-04
chr3   24889        Up 0.00339 0.00543     1.22e-04  1.22e-04
chr9   26363        Up 0.00365 0.00543     6.43e-05  1.15e-04
chr8   29208        Up 0.00369 0.00543     5.90e-05  1.15e-04
chr11  35826        Up 0.00371 0.00543     4.70e-05  1.15e-04
chr13  22202        Up 0.00372 0.00543     8.61e-05  1.15e-04
chr14  18362        Up 0.00377 0.00543     6.85e-05  1.15e-04
chr12  20927        Up 0.00390 0.00543     8.22e-05  1.15e-04
chr1   30567        Up 0.00400 0.00543     7.50e-05  1.15e-04
chr6   24880        Up 0.00412 0.00543     1.09e-04  1.15e-04
chr5   37115        Up 0.00436 0.00543     8.93e-05  1.15e-04
chr19  15758        Up 0.00460 0.00543     8.50e-05  1.15e-04
chr16  16025        Up 0.00462 0.00543     6.52e-05  1.15e-04
chr4   34681        Up 0.00532 0.00591     8.54e-05  1.15e-04
chr17  23812        Up 0.01027 0.01082     7.03e-05  1.15e-04
chrX   16562        Up 0.93464 0.93464     4.27e-06  8.55e-05

This shows that methylation increases overall in committed luminal cells for every chromosome except for Chromosome X. This is consistent with the expectation that methylation is associated with silencing of genes not needed in a committed lineage. The column heading NGenes gives the number of CpG loci in each chromosome.

The results for Chromosome X are interesting. The mixed P-value shows that the X chromosome is actually enriched for DM CpGs, but the direction of change is not consistent with hyper and hypomethylated CpGs balancing each other.

### Global methylation patterns around TSS

Now we explore methylation patterns to relative to gene TSSs. The coefficients of the fitted model fit record methylation levels at each CpG in each cell population. We now relate methylation in the P6 (luminal) population versus distance to the nearest TSS:

> i <- abs(fit$genes$Distance) < 20000
> lo <- lowess(fit$genes$Distance[i], fit$coefficient[i,"P6"], f=0.3)
> plot(lo, type="l", xlab="Distance to TSS", ylab="Logit Methylation Level",
+       main="P6 (Luminal)")
> abline(h=0, lty=2)
> abline(v=0, lty=2)

**Figure 3.**
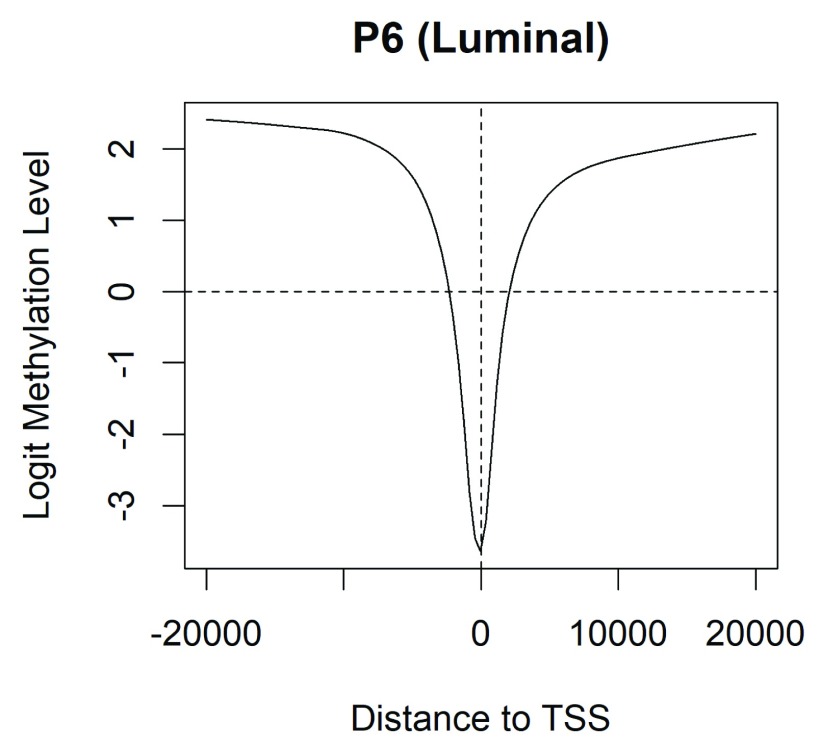
Methylation level by distance to the nearest gene TSS. Results are averaged over all genes. Negative distances are downstream of TSS.

Figure 3 shows that CpGs near a TSS tend to be unmethylated but CpGs elsewhere are predominately methylated. Cell populations P7 and P8 show the same patterns.

Now we examine the methylation changes stored in lrt and relate these to TSS position.

> i <- abs(lrt$genes$Distance) < 80000
> lo <- lowess(lrt$genes$Distance[i], lrt$table$logFC[i], f=0.3)
> plot(lo, type="l", xlab="Distance to TSS", ylab="Change in Methylation",
+       main="Luminal vs Basal")
> abline(v=0, lty=2, col="grey")

**Figure 4.**
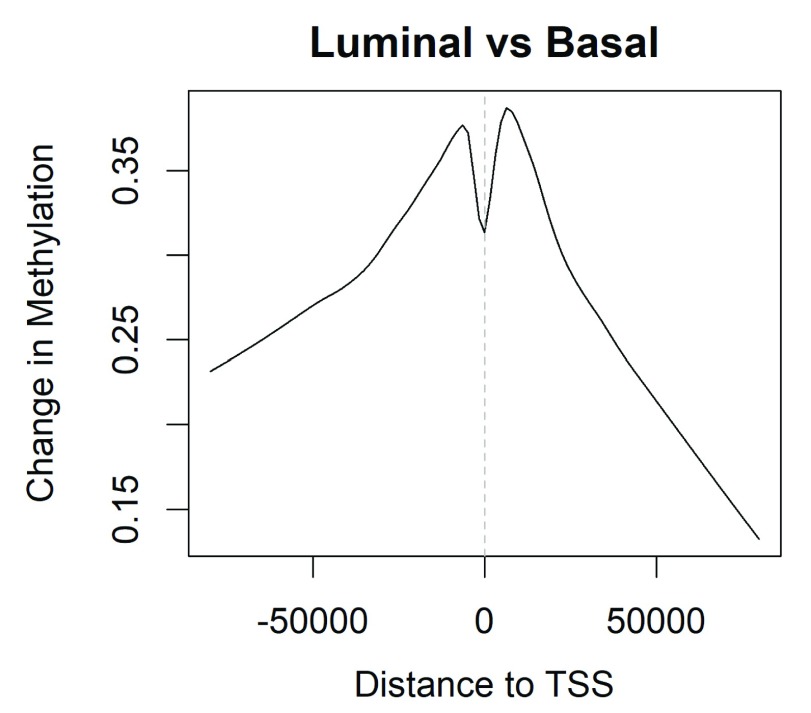
Average methylation change by genomic position relative to TSS. Changes are averaged over all genes. Negative distances are downstream of TSS. Vertical axis shows base 2 logit differences.

Figure 4 shows that methylation tends to be added around the TSS in luminal vs basal cells. CpGs further from TSS tend to be unchanged.

## Differential methylation in gene promoters

### Pre-defined gene promoters

The majority of CpGs are methylated in mammals. On the other hand, unmethylated CpGs tend to group into clusters of CpG islands, which are often enriched in gene promoters. CpG methylation in promoter regions is often associated with silencing of transcription and gene expression ^3^. Therefore it is of great biological interest to examine the methylation level within the gene promoter regions.

For simplicity, we define the promoter of a gene as the region from 2kb upstream to 1kb downstream of the transcription start site of that gene.

> InPromoter <- yall$genes$Distance >= -1000 & yall$genes$Distance <= 2000

We subset the CpGs to those contained in a promoter region:

> yIP <- yall[InPromoter,,keep.lib.sizes=FALSE]

### Summarizing counts in promoter regions

One simple and effective way to conduct a gene-orientated analysis of the methylation changes is to collapse all the CpGs in each promotor into one locus, i.e., to compute the total number of methylated and unmethylated reads within each promoter. This strategy focuses on the aggregate methylation level within each promoter and allows us to test for an overall increase or decrease in methylation for each gene.

First we compute the total counts for each gene promoter:

> ypr <- rowsum(yIP, yIP$genes$EntrezID, reorder=FALSE)
> ypr$genes$EntrezID <- NULL

The rowsum function here operated on the DGEList object yIP and produces a new DGEList object ypr with a row for each EntrezID. The integer matrix ypr$counts contains the total numbers of methylated and unmethylated CpGs observed within the promoter of each gene. Same as before, ypr$counts has 12 columns, two for each sample. The odd-numbered columns contain the numbers of methylated Cs, whereas the even-numbered columns contain the numbers of unmethylated Cs. The only difference is that each row of ypr$counts now represents a gene promoter instead of an individual CpG site.

### Filtering to remove low counts

Filtering is performed in the same way as before. We sum up the read counts of both methylated and unmethylated Cs at each gene promoter within each sample.

> Coveragepr <- ypr$counts[,Methylation=="Me"] +
+               ypr$counts[,Methylation=="Un"]

Since each row represents a 3,000-bps-wide promoter region that contains multiple CpG sites, we would expect less filtering than before.

> keeppr <- rowSums(Coverageprm >= 10) == 6
> table(keeppr)

keeppr
FALSE  TRUE
 1949 16837

> ypr <- ypr[keeppr,,keep.lib.sizes=FALSE]

Same as before, we do not perform normalization but set the library sizes for each sample to be the average of the total read counts for the methylated and unmethylated libraries.

> TotalLibSizepr <- 0.5*ypr$samples$lib.size[Methylation=="Me"] +
+                     0.5*ypr$samples$lib.size[Methylation=="Un"]
> ypr$samples$lib.size <- rep(TotalLibSizepr, each=2)
> ypr$samples

         group lib.size norm.factors
P6_1-Me    P6 11620642            1
P6_1-Un    P6 11620642            1
P6_4-Me    P7 11740775            1
P6_4-Un    P7 11740775            1
P7_2-Me    P8  4781385            1
P7_2-Un    P8  4781385            1
P7_5-Me    P6 10426283            1
P7_5-Un    P6 10426283            1
P8_3-Me    P7  3854829            1
P8_3-Un    P7  3854829            1
P8_6-Me    P8  3329192            1
P8_6-Un    P8  3329192            1

### Exploring differences between samples

Same as before, we measure the methylation levels of gene promoter regions using M-values. A prior count of 2 is added to the calculation to avoid undefined values and to reduce the variability of M-values for gene promoters with low counts. Then a MDS plot is produced to examine the overall differences between the methylation levels of the different samples.

> Me <- ypr$counts[, Methylation=="Me"]
> Un <- ypr$counts[, Methylation=="Un"]
> M2 <- log2(Me + 2) - log2(Un + 2)
> colnames(M2) <- Sample
> plotMDS(M2)

**Figure 5.**
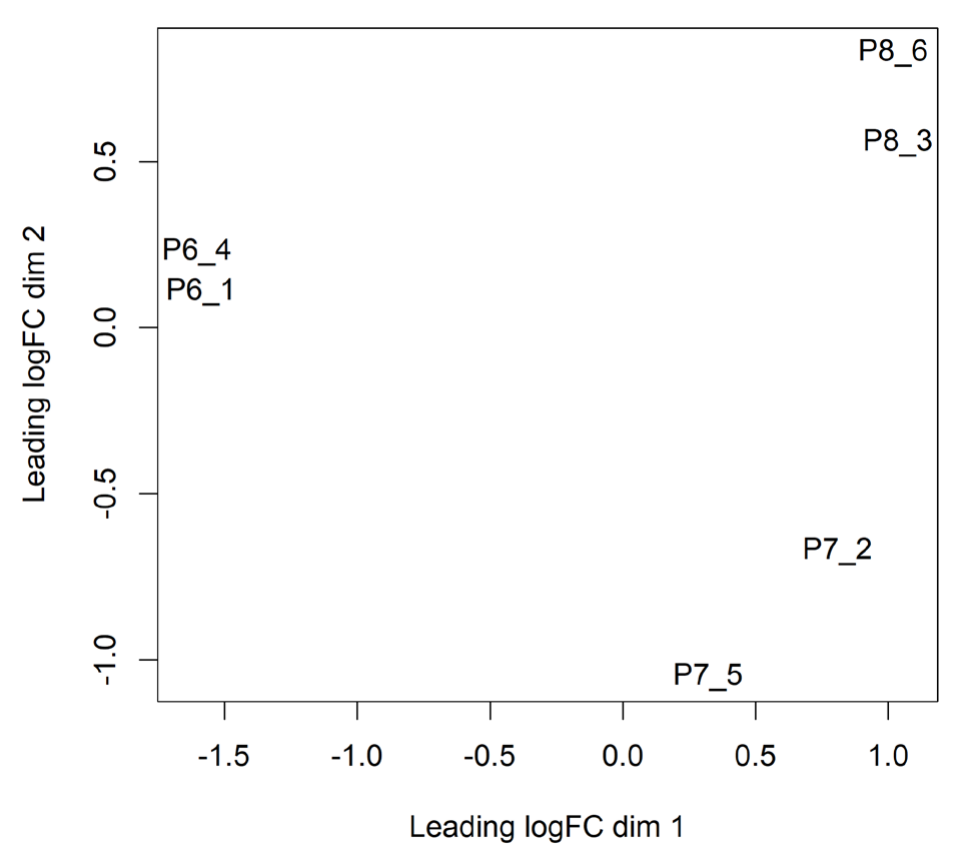
MDS plot showing differences in methylation profiles at gene promoters. Basal and luminal cell populations are well separated by the first dimension.

The resulting Figure 5 shows that the two replicate samples from the luminal population (P6) are well separated from the four replicate samples from the basal population (P7 and P8).

### Dispersion estimation

We estimate the NB dispersions using the estimateDisp function in *edgeR*. For the same reason, we do not consider a mean-dependent dispersion trend as we normally would for RNA-seq data.

> ypr <- estimateDisp(ypr, design, trend="none")
> ypr$common.dispersion

[1] 0.0304

> ypr$prior.df

[1] 10.7

**Figure 6.**
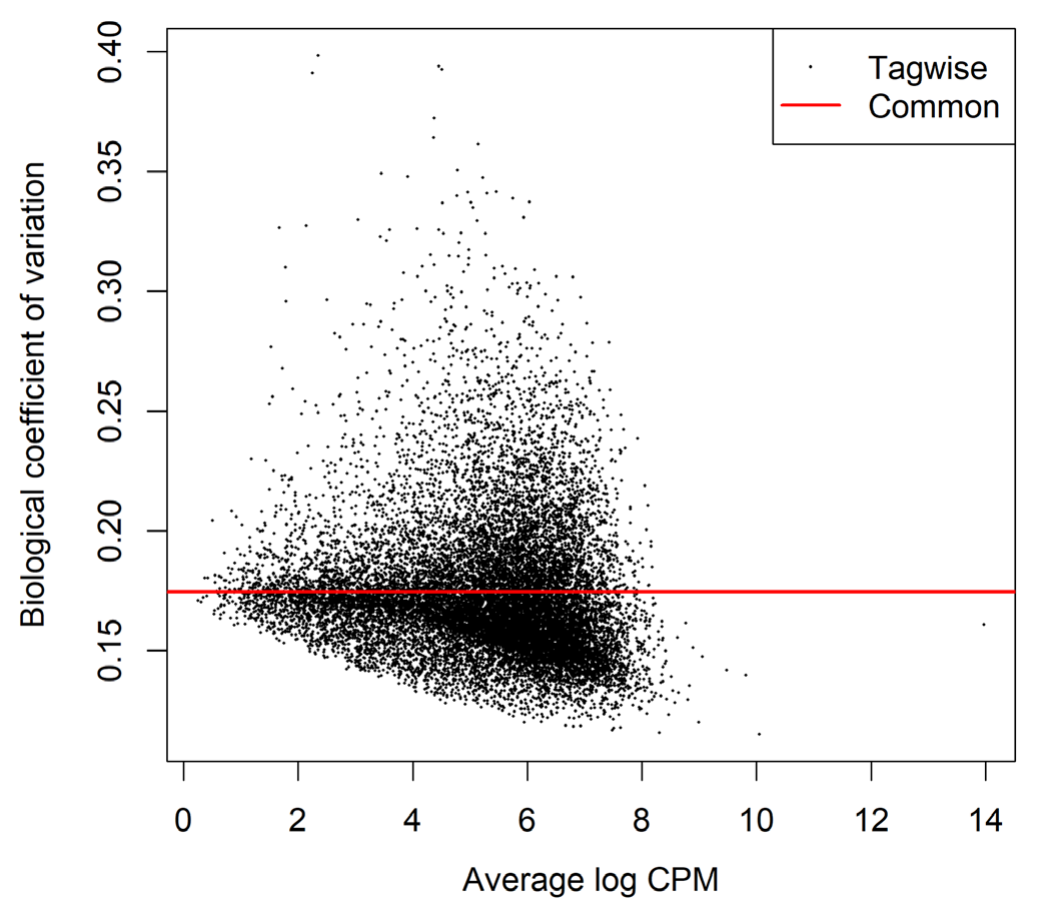
Scatterplot of the biological coefficient of variation (BCV) against the average abundance of each gene. The plot shows the square-root estimates of the common and tagwise NB dispersions.

The dispersion estimates ( *ϕ _g_* ) can be visualized with a BCV plot (see Figure 6):

> plotBCV(ypr)

### Testing for differential methylation in gene promoters

We first fit NB GLMs for all the gene promoters using glmFit.

> fitpr <- glmFit(ypr, design)

Then we can proceed to testing for differential methylation in gene promoter regions between different populations. Suppose the comparison of interest is the same as before. The same contrast can be used for the testing.

> lrtpr <- glmLRT(fitpr, contrast=contr)

The top set of most differentially methylated gene promoters can be viewed with topTags:

> topTags(lrtpr, n=20)

Coefficient:  1*P6 -0.5*P7 -0.5*P8
            Chr        Symbol Strand logFC logCPM  LR   PValue      FDR
16924      chr5          Lnx1      - -6.85   5.34 313 4.92e-70 8.29e-66
238161    chr12         Akap6      + -5.26   4.16 248 6.55e-56 5.51e-52
64082     chr16        Popdc2      +  4.91   5.94 225 7.96e-51 4.47e-47
11601      chr8        Angpt2      -  5.58   3.40 208 3.45e-47 1.45e-43
108168987  chr4       Gm13205      -  4.37   7.13 196 1.57e-44 5.29e-41
12740      chr5         Cldn4      - -5.56   5.26 189 6.49e-43 1.82e-39
16669     chr11         Krt19      - -5.05   6.58 187 1.65e-42 3.96e-39
73644     chr12 2210039B01Rik      + -4.31   5.36 180 6.00e-41 1.26e-37
387514     chr6      Tas2r143      +  4.52   4.59 177 2.10e-40 3.94e-37
321019    chr14        Gpr183      -  5.86   2.92 152 5.36e-35 9.02e-32
76509      chr9         Plet1      + -6.00   2.45 146 1.43e-33 2.18e-30
100043766  chr2       Gm14057      -  5.40   3.74 145 2.04e-33 2.87e-30
68386      chr2 0610039K10Rik      + -4.43   6.58 143 4.70e-33 6.09e-30
110308    chr15          Krt5      -  4.09   3.62 140 2.60e-32 3.13e-29
59091      chr2          Jph2      -  3.56   4.70 139 3.46e-32 3.89e-29
244198     chr7        Olfml1      +  5.91   3.36 133 1.04e-30 1.10e-27
18291      chr6         Nobox      -  4.75   3.37 130 5.22e-30 5.17e-27
21345      chr9         Tagln      -  4.62   4.25 127 1.54e-29 1.44e-26
653016    chr17          Mymx      -  2.75   5.69 127 1.71e-29 1.52e-26
229277     chr3        Stoml3      +  4.85   2.73 126 3.09e-29 2.60e-26

Here positive log-fold-changes represent gene promoters that have higher methylation level in the luminal population compared to the basal population. The Benjamini-Hochberg multiple testing correction is applied to control the false discovery rate (FDR).

The total number of DM gene promoters identified at an FDR of 5% can be shown with decideTests. There are in fact about 1,200 differentially methylated gene promoters in this comparison:

> summary(decideTests(lrtpr))

        1*P6 -0.5*P7 -0.5*P8
Down                    368
NotSig                15627
Up                      842

For future reference we make a dataframe of all the DM genes:

> topME <- topTags(lrtpr, n=Inf, p=0.05)$table
> dim(topME)

[1] 1210    8

**Figure 7.**
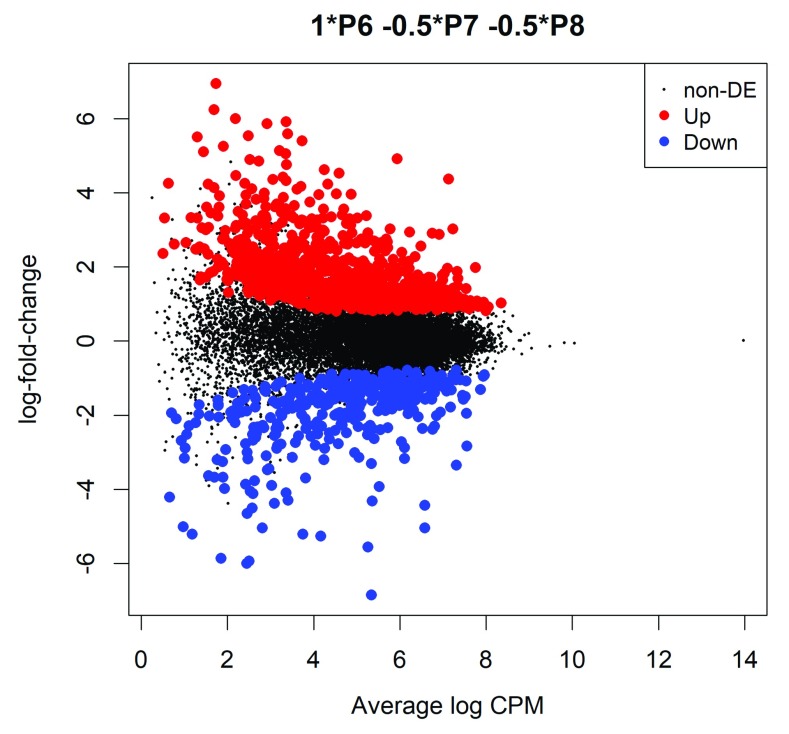
MD plot showing the log-fold-change of the methylation level and average abundance of CpG sites in each gene promoter. Significantly hyper and hypomethylated gene promoters are highlighted in red and blue, respectively.

The differential methylation results can be visualized with an MD plot (see Figure 7):

> plotMD(lrtpr)

## Correlate with RNA-seq profiles

### RNA-seq profiles of mouse epithelium luminal and basal cells

To explore whether hypermethylation of promoter regions is associated with repressed gene expression, we relate the differential methylation results to differential expression results from RNA-seq for similar cell populations. The RNA-seq data used here is from a study of the epithelial cell lineage in the mouse mammary gland, in which the expression profiles were generated from basal stem-cell enriched cells and committed luminal cells in the mammary glands of virgin, pregnant and lactating mice ^40^. The complete differential expression analysis of the data is described in Chen *et al*. ^32^.

The RNA-seq data is stored as a DGEList object y_rna and saved in a RData file rna.RData. The object y_rna contains the count matrix, sample information, gene annotation, design matrix and dispersion estimates of the RNA-seq data. The gene filtering, normalization and dispersion estimation were performed in the same way as described in Chen *et al*. ^32^. The rna.RData file is available for download at http://bioinf.wehi.edu.au/edgeR/F1000Research2017.

We load the RData file:

> load("rna.RData")
> dim(y_rna)

[1] 15641    12

> y_rna$samples

                group lib.size norm.factors
MCL1.DG    B.virgin 23137472        1.235
MCL1.DH    B.virgin 21687755        1.213
MCL1.DI  B.pregnant 23974787        1.125
MCL1.DJ  B.pregnant 22545375        1.069
MCL1.DK B.lactating 21420532        1.036
MCL1.DL B.lactating 19916685        1.087
MCL1.LA    L.virgin 20273585        1.370
MCL1.LB    L.virgin 21568458        1.368
MCL1.LC  L.pregnant 22117517        1.006
MCL1.LD  L.pregnant 21877287        0.924
MCL1.LE L.lactating 24657903        0.529
MCL1.LF L.lactating 24600304        0.535

We keep only the genes that are also included in our methylation analysis:

> haveME <- row.names(y_rna) %in% row.names(ypr)
> y_rna <- y_rna[haveME,]
> dim(y_rna)

[1] 13210 12


We assess differential expression between the luminal and basal virgin samples:

> fitrna <- glmFit(y_rna)
> Contrastrna <- makeContrasts(L.virgin-B.virgin, levels=y_rna$design)
> lrtrna <- glmLRT(fitrna, contrast=Contrastrna)

### Correlate methylation and expression

We can add the expression log-fold-changes to our methylation results:

> topME$logFC.RNA <- lrtrna$table[row.names(topME),"logFC"]
> topME[1:30,c("Symbol","logFC","logFC.RNA")]

                 Symbol logFC logFC.RNA
16924              Lnx1 -6.85    2.2748
238161            Akap6 -5.26   -3.2258
64082            Popdc2  4.91   -7.6743
11601            Angpt2  5.58   -2.1021
108168987       Gm13205  4.37        NA
12740             Cldn4 -5.56    5.0985
16669             Krt19 -5.05    4.5266
73644     2210039B01Rik -4.31        NA
387514         Tas2r143  4.52   -3.1034
321019           Gpr183  5.86   -1.9598
76509             Plet1 -6.00    4.8335
100043766       Gm14057  5.40   -0.8902
68386     0610039K10Rik -4.43    2.1650
110308             Krt5  4.09   -8.9403
59091              Jph2  3.56   -7.2598
244198           Olfml1  5.91        NA
18291             Nobox  4.75        NA
21345             Tagln  4.62   -6.4003
653016             Mymx  2.75        NA
229277           Stoml3  4.85        NA
16846               Lep  3.96   -6.1556
231253    9130230L23Rik -5.21    3.9109
16012            Igfbp6  3.02   -2.4216
675921           Tnk2os -3.69    2.0039
11529              Adh7  5.53   -0.0508
100526468       Mir3063  3.56        NA
78896     1500015O10Rik  6.94   -6.6807
217143           Gpr179  5.05   -2.9424
17242               Mdk  2.93   -4.5784
100526559       Mir3103  6.24        NA




The negative correlation between methylation and expression is immediately apparent. Of the top 30 DM genes, eight have NA expression fold-changes because the genes were not expressed at a high enough level to be included in the analysis. Of the rest, all but Akap6 have methylation and expression logFCs of opposite signs.

**Figure 8.**
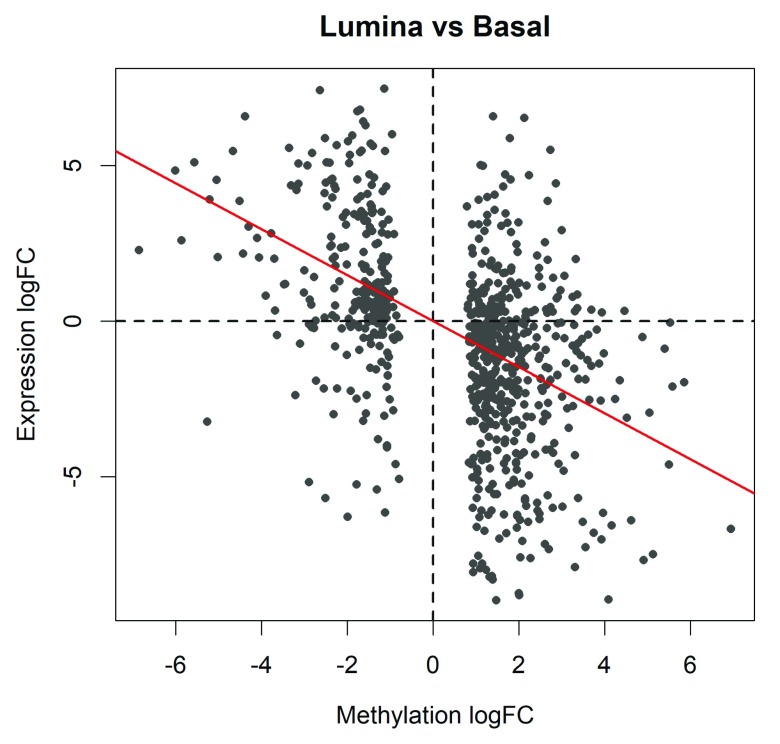
Scatter plot of the log-fold-changes of methylation levels in gene promoters (x-axis) vs the log fold-changes of gene expression (y-axis). The plot shows results for the genes of which the promoters are significantly differentially methylated between basal and luminal. The red line shows the least squares line with zero intercept. A strong negative correlation is observed.

We can explore this correlation further by plotting the expression logFC vs the methylation logFC for all DM genes (see Figure 8):

> plot(topME$logFC, topME$logFC.RNA, main="Lumina vs Basal",
+      xlab="Methylation logFC", ylab="Expression logFC",
+      pch=16, cex=0.8, col="gray30")
> abline(h=0, v=0, col="gray10", lty=2, lwd=2)

The horizontal axis of the scatterplot shows the log-fold-change in methylation level for each gene promoter while the vertical axis shows the log-fold-change in gene expression. To assess the correlation, we fit a least squares regression line through the origin and compute the P-value:

> RNAvsME <- lm(topME$logFC.RNA ~ 0 + topME$logFC)
> coef(summary(RNAvsME))

            Estimate Std. Error t value Pr(>|t|)
topME$logFC   -0.738     0.0472   -15.7 6.17e-48

> abline(RNAvsME, col="red", lwd=2) 



The negative association is highly significant ( *P* = 6 *×* 10 ^*−*48^). The last line of code adds the regression line to the plot ( Figure 8).

### Gene set testing

The correlation in Figure 8 is convincing, but the above P-value computation assumes that genes are statistically independent of one another. We can perform a gene set test to get around this assumption.

First we make a dataframe of the logFCs of the DM genes, keeping only the genes for which we have RNA-seq results:

> ME <- data.frame(ID=row.names(topME), logFC=topME$logFC, stringsAsFactors=FALSE)
> inRNA <- ME$ID %in% row.names(y_rna)
> ME <- ME[inRNA,]

Then we test whether the DM genes are up or down-regulated using a “fry” gene set test. This analysis weights the DM genes by their methylation logFCs so that the test evaluates whether the expression changes are positively or negatively associated with the methylation changes:

> fry(y_rna, index=ME, contrast=Contrastrna)

     NGenes Direction   PValue PValue.Mixed
set1    740      Down 1.28e-09        5e-11


The result Down in the Direction column indicates negative correlation between the methylation and expression changes. The small PValue confirms a highly significant result.

**Figure 9.**
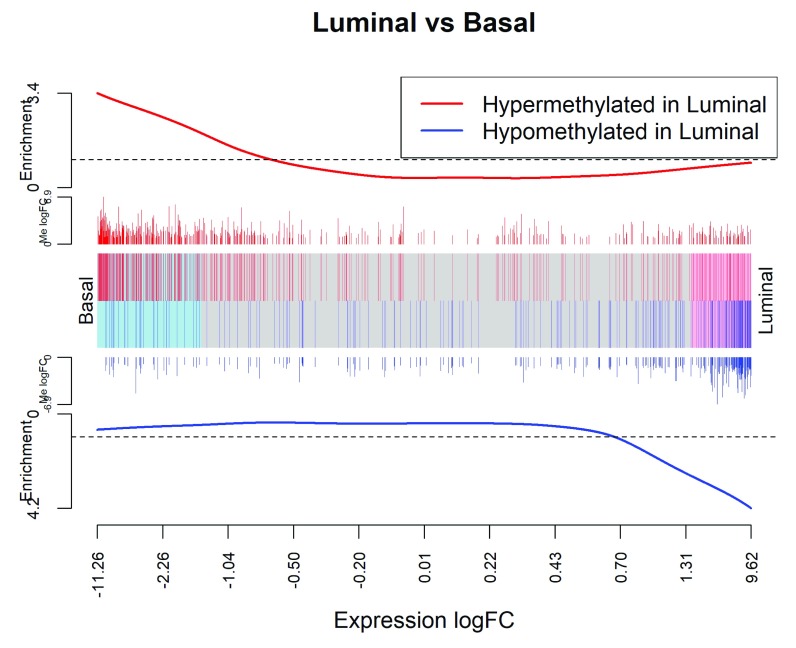
Barcode plot showing strong negative correlation between expression changes and methylation changes when luminal cells are compared to basal. The plot is analogous to Figure 8 with the DM genes now displayed as gene sets and the methylation logFCs now displayed as gene weights.

We can visualize the gene set test result with a barcode plot (see Figure 9):

> logFC.ME <- rep_len(0,nrow(y_rna))
> names(logFC.ME) <- row.names(y_rna)
> logFC.ME[ME$ID] <- ME$logFC
> barcodeplot(lrtrna$table$logFC, gene.weights=logFC.ME,
+               labels=c("Basal","Luminal"), main="Luminal vs Basal",
+               xlab="Expression logFC", weights.label="Me logFC")
> legend("topright", col=c("red","blue"), lty=1, lwd=2,
+         legend=c("Hypermethylated in Luminal", "Hypomethylated in Luminal"))

In the barcode plot, genes are sorted left to right from most down-regulated to most up-regulated in luminal vs basal. The x-axis shows the expression log2-fold-change. The vertical red bars indicate genes hypermethylated in luminal and vertical blue bars indicate genes hypomethylated in luminal. The variable-height vertical bars show the methylation log-fold-changes. The red and blue worms measure relative enrichment, showing that hypermethylation is associated with decreased regulation and hypomethylation is associated with up-regulation. In other words, there is a strong negative association between methylation of promoter regions and expression of the corresponding genes.

## Discussion

This article has presented a complete start to finish analysis of an RRBS dataset from our own practice. The analysis demonstrates how BS-seq data can be analyzed using software designed for RNA-seq, thus benefiting from a large reservoir of highly-developed RNA-seq methodology. At first sight, BS-seq data is fundamentally different to RNA-seq because of the need to focus on the *proportion* of methylated reads at each locus rather than on the absolute number of reads at each locus. The link is achieved by conducting the statistical inference conditional on the read coverage at each locus, and this in turn is achieved by including sample-specific locus effects in the linear model.

We have concentrated on analysing methylation changes for pre-defined genomic regions. At the highest resolution, conducted differential methylation tests for each distinct CpG site. Our main analysis was gene-level, whereby we aggregated counts over a putative promoter region around the TSS for each gene. This gives a “big picture” analysis and facilities a correlation of DM results with differential expression results from RNA-seq for the same cell populations. We also illustrated the use of gene set tests to examine whether there were overall methylation changes at the whole chromosome level.

The analysis presented here was designed for reduced representation BS-seq and was tuned to our own research interests. While the same analysis approach could be fruitfully applied to WGBS data, researchers with WGBS data may also want to discover DMRs *de novo* without the use of gene annotation or pre-specified genomic regions, something we haven’t explored in this article. The *edgeR* approach presented here could in principle be extended to discover DMRs in a *de novo* fashion using similar methods to those developed for ChIP-seq data ^41– 43^.

## Packages used

This workflow depends on various packages from version 3.7 of the Bioconductor project, running on R version 3.5.0 or higher. For all the code to work as presented, *edgeR* 3.22.2 or later is required. A complete list of the packages used for this workflow is shown below:

> sessionInfo()

R version 3.5.1 (2018-07-02)
Platform: x86_64-w64-mingw32/x64 (64-bit)
Running under: Windows 10 x64 (build 15063)

Matrix products: default

locale:
[1] LC_COLLATE=English_Australia.1252  LC_CTYPE=English_Australia.1252
[3] LC_MONETARY=English_Australia.1252 LC_NUMERIC=C
[5] LC_TIME=English_Australia.1252

attached base packages:
[1] stats     graphics  grDevices utils     datasets methods base

other attached packages:
[1] edgeR_3.22.5 limma_3.36.5 knitr_1.20

loaded via a namespace (and not attached):
 [1] Rcpp_0.12.18         AnnotationDbi_1.42.1 magrittr_1.5
 [4] IRanges_2.14.11      BiocGenerics_0.26.0  hms_0.4.2
 [7] bit_1.1-14           lattice_0.20-35      R6_2.2.2
[10] rlang_0.2.2          blob_1.1.1           stringr_1.3.1
[13] highr_0.7            tools_3.5.1          parallel_3.5.1
[16] grid_3.5.1           Biobase_2.40.0       DBI_1.0.0
[19] bit64_0.9-7          digest_0.6.17        tibble_1.4.2
[22] crayon_1.3.4         org.Mm.eg.db_3.6.0   readr_1.1.1
[25] S4Vectors_0.18.3     codetools_0.2-15     memoise_1.1.0
[28] evaluate_0.11        RSQLite_2.1.1        stringi_1.1.7
[31] compiler_3.5.1       pillar_1.3.0         stats4_3.5.1
[34] locfit_1.5-9.1       pkgconfig_2.0.2



## Data and software availability

All software packages used in this workflows are publicly available as part of Bioconductor 3.7. The data and analysis code used in this workflow are available from http://bioinf.wehi.edu.au/edgeR/F1000Research2017. The data and analysis code as at time of publication of this article has been archived at http://doi.org/10.5281/zenodo.1052870 ^44^.
