# Supplementary material for: Differential methylation analysis of reduced representation bisulfite sequencing experiments using edgeR
Source: F1000Res. 2018 Oct 8;6:2055. Originally published 2017 Nov 28. [Version 2] doi: 10.12688/f1000research.13196.2 (PMC5747346; doi:10.12688/f1000research.13196.2)
Supplement: Peer review history

# Peer review: Referee response for version 1

**DOI:** 10.5256/f1000research.14317.r28485

**Published:** 2017-12-20

**Authors:** Peter F. Hickey

**Affiliations:** 1 Department of Biostatistics, Johns Hopkins University, Baltimore, MD, USA

Chen *et al.* propose a novel use of negative binomial generalized linear models (GLMs), as implemented in the **edgeR** software, to test for differential methylation from bisulfite-sequencing data, particularly reduced representation bisulfite-sequencing (RRBS). By leveraging the existing **edgeR** software, a popular tool in the analysis of diffential gene expression analysis from RNA-seq data, this method is immediately able to handle complex experimental designs and integrates with downstream analysis tools such as gene set tests provided by the **limma **software. The paper is well-written and I was able to reproduce the authors’ analysis. It will be a useful workflow for people needing to develop an analysis of RRBS data.

 Like Simon Andrews, I initially struggled a little with some of the detail of the method itself. The method’s elegance and power, like all those based on (generalized) linear models, is driven by careful formulation of the design matrix and the choice of contrasts. Necessarily, the design matrix for analysing bisulfite-sequencing data is more complex than that used to analyse RNA-seq data from an identical experimental design. As I know the authors are well aware, getting the design matrix and contrasts correct is 95% of the battle for most people analysing data with **edgeR** and **limma**. I will explain my concerns below (many are the same as raised by Simon in his review).

**Main points**

- p4: The initial example has no replicates. Since the method is designed for “any BS-seq data that includes some replication”, should this example include replicates? I appreciate the desire to keep the initial example simple (especially in light of my next comment).
- p4: I initially found the design matrix confusing. In fact, I had the same reaction/interpretation as Simon Andrews 4th comment: “In the small example description you say that A_MvsU estimates the log ratio for Sample1, but it wasn’t clear to me why this would apply to only Sample 1 since the factor has a 1 against the meth count for both samples 1 and 2”. I had to manually check a few quantities to convince myself, e.g., to rounding error, `coef(fit)[, "A_MvsU"]` is `logit((2 + prior.count) / (2 + prior.count + 12 + prior.count)`, where `prior.count = 0.125`. Because so much depends on constructing the appropriate design matrix, this description/section may warrant further explanation (e.g., comparing to some manually computed quantities).
- Like James MacDonald, although the code was clearly written, I was a little surprised that it didn’t use more consistent integration with existing Bioconductor packages and data structures. To add to his example, almost all the work in the section ‘Reading in the data’ can be achieved with `bsseq::read.bismark(fn)`, which will: read in an arbitrary number of Bismark `.cov.gz` files, appropriately combine samples with different sets of CpGs, and return a *SummarizedExperiment*-derived object (a *BSseq* instance) which could readily be used to construct the *DGEList* used in the analysis. In my experience, loading the data and combining different sets of loci is a step fraught with danger of hard-to-track-down errors, so it may be better to advise workflow users to use a fairly well-tested function. Full disclosure: I am the author of `bsseq::read.bismark()`.
- p14: The aggregation of CpGs to promoters may lead to surprising results. An (extreme) example: the first half of a promoter is methylated in one condition and unmethylated in the other, and vice versa for the second half of the promoter. In aggregate over the promoter the proportion of methylated CpGs may be similar in both conditions, yet this promoter is clearly differentially methylated. I think a note encouraging workflow users to think carefully about their hypothesis when doing this form of aggregation is warranted.

Minor points

- p1: “The most commonly used technology of studying DNA methylation is bisulfite sequencing (BS-seq)”. The Illumina 27k/450k/EPIC microarrays are the most commonly used ‘genome-wide’ assays for studying DNA methylation. However, (whole genome) BS-seq is arguably the gold standard genome-wide assay.
- p3: I think there’s some confusion about CpGs and CpG islands (CGI). Approximately 0.9% of dinucleotides in the human genome (hg19) are CpGs, and approximately 0.7% of the genome is a CGI (using UCSC CGIs, which is not the only definition but perhaps the standard); see code below:

```R

 library(BSgenome.Hsapiens.UCSC.hg19)

 hg19_size <- sum(as.numeric(seqlengths(BSgenome.Hsapiens.UCSC.hg19)[

   paste0("chr", c(1:22, "X", "Y"))]))

 # CpGs on chr1-22,chrX,chrY in hg19

 n_CpGs <- Reduce(sum, bsapply(BSParams = new("BSParams",

                                              X = BSgenome.Hsapiens.UCSC.hg19,

                                              FUN = countPattern,

                                              exclude = c("M", "_")),

                               pattern = "CG"))

 100 * n_CpGs / hg19_size

 # CGIs in hg19                              

 library(rtracklayer)

 my_session <- browserSession("UCSC")

 genome(my_session) <- "hg19"

 cgi <- track(ucscTableQuery(my_session, track = "cpgIslandExt"))

 sum(width(cgi)) / hg19_size

 ```

- p3: Possible type, “with *a* large genome”
- p3: “WGBS is more suitable for studies where all CpG islands or promoters across the entire genome are of interest.” Might also add ‘distal regulatory elements’ and CG-poor regions (RRBS targets CG-rich regions of the genome).
- p3: **BSmooth** (implemented in **bsseq**) doesn’t use Empirical Bayes although it does use **limma** for linear regression
- p4: Missing a `library(edgeR)` in order for the code to work
- p4: There’s an extra parenthesis at the end of line 2 when constructing `dimnames(counts)`
- p4: The authors note that the method is “especially appropriate for RRBS data”. Is the main challenge for running on WGBS data that of computational resources?
- p5: Typo, “mythlyated” should be “methylated”
- Table 1: Condition should be ‘A’ or ‘B’ instead of ‘1’ or ‘2’
- p6: Was Bowtie1 or Bowtie2 used as the Bismark backend for the mouse data?
- p8: The filtering step removes almost 90% of CpGs. Is this unavoidable, e.g., due to low sequencing coverage of these samples, or might the filtering be relaxed?
- Figure 1: Any thoughts for why the P8_6 sample is rather separated from the other Basal samples along dim2 of the MDS plot?
- Figure 2: What is the meaning of ‘average abundance of each CpG site’? Is ‘abundance’ interpretable as ‘sequencing depth’?
- p16: Possible typo, “Suppose the comparison of interest is *the* same as before”
- p22: In the DNA methylation literature, ‘up-methylated’ is typically called ‘hypermethylated’ and ‘down-methylated’ is typically called ‘hypomethylated’.

I have read this submission. I believe that I have an appropriate level of expertise to confirm that it is of an acceptable scientific standard.

# Response

**Published:** 2018-09-30

**Authors:** Gordon Smyth

**Affiliations:** Walter and Eliza Hall Institute of Medical Research, Australia

Thank you for your thoughtful and positive comments on our article.

We particularly wish to thank you for your comments on the introductory material, which we have rewritten to take account of all your suggestions. We have specified the version of Bowtie, adopted the “hyper” and “hypo” terminology and made all the other corrections.

In this revision we have added a lot of new material to the expository section, including an example with replicates, while retaining the first example without replicates. The purpose of the first example is to introduce the key mathematical idea that underlies our article, which is the use of linear model terms to account for total read coverage at each CpG, and to relate this to the more familiar beta-binomial modeling approach. This idea is already manifest in the very small dataset without replicates, and so that is the most fundamental context in which to exposit the idea. The need for replicates relates to dispersion estimation, which is a separate issue. For pedagogic purposes it is best to isolate these concepts separately.

We now demonstrate in the first example how the logFC is computed in complete arithmetic detail. We have switched the toy examples to use the group-mean parametrization instead of the treatment-parametrization so that the computation of logFC is explicit rather than implicit. See also our reply to Simon Andrews.

We have introduced a new function modelMatrixMeth() to automate the construction of the design matrix. From a user point of view, the design matrix is now made exactly as it would be for an RNA-seq analysis, with the sample effects added automatically. We have also added a new section “Analogy with paired-samples expression analyses” to show that the entire linear modeling approach is equivalent to an (already well understood) type of RNA-seq experiment.

The purpose of our article is to show that methylation data can be analyzed exactly like RNA-seq and we use the same packages and data structures as our earlier RNA-seq workflow article (Chen et al, 2017). Adding extra packages and data structures would complicate rather than help our workflow.

We are aware of your read.bismark() function in the bbseq package. When run on our data, read.bismark() produces a number of alarming warnings suggesting that incorrect genome annotation has been used for some of the samples. These warnings are false alarms, but would worry users, so we would have to either suppress them or explain them to readers, were we to adopt read.bismark.

We are also aware of the readBismark() function in the BiSeq package and the methRead() function in the methylKit package. It seems that every Bioconductor package that analyses BS-seq has its own read function. The functions read the methylation data into a binomial format, whereas the whole point of our article is to show that the methylated and unmethylated counts can be analysed as they are instead of doing a binomial analysis. All produce data structures that are incompatible with edgeR. We could wrangle the data formats back to a DGEList, but the end result would be code that is no shorter or simpler than our original plain base R code. In fact the code would be harder to follow because of the extra packages and data structures involved.

It seems unfortunate that the Bioconductor methylation packages all create their own read functions from scratch in order to create their own specialist S4 objects. Each defines a complex S4 class, unique to that one package. If Bioconductor provided a fast, light-weight, base-R style function to read Bismark files, then all the packages could use that as building block instead of re-writing from scratch.

In this revision, we have decided to create a new function readBismark2DGE() to read the Bismark files directly into a DGEList object, substantially simplifying our workflow for users. The new function is faster than any existing options and over 3 times as fast as read.bismark() on the workflow data. The new function creates simple list output with minimal processing and so serves the purpose of a light-weight base-R style function.

You comment on aggregation over promoters and guidance about hypotheses. We have added more text to clarify that aggregation of counts over gene promoters tests for overall changes only, but we think that users would have expected this already and shouldn’t be surprised.

The thought example you give is indeed extreme and does not match real that we have seen. Even if such a phenomenon could occur, our workflow includes a CpG-level analysis, so readers can easily check for CpGs changing in different directions within a promoter if that is of interest to them. We did this check ourselves and found that for 99% of genes whose promoters contain any DM CpGs, all the DM CpGs change in the same direction. In almost all the remaining cases, only one CpG changes in the “wrong” direction. The only exception is Mir1298 on Chromosome X that has two CpGs changing in the other direction to the majority.

Even if there were opposing effects within a few gene promoters, it wouldn’t invalidate our aggregate analysis. It would simply mean that exceptional genes would have to analyzed and interpreted individually to understand the fully story, and we see no problem with that.

We think there is a role for both aggregate analyses and high-resolution analyses of DNA methylation. However we think that aggregate analyses are under-used in the literature and should be encouraged more than at present. We think that genomic analysts sometimes try to accommodate too much complexity, leading to analyses that lack statistical power. This in turn leads to the danger of cherry picking, possibly over-interpreting results that might not be reproducible. Aggregate analyses ignore some of the complexity, but can lead to more reliable and interpretable “big picture” messages. In this article, our promoter region aggregated analysis leads to a clean and reliable correlation of methylation with RNA-seq results, a correlation that is more difficult to achieve by other means.

Our remark that our method is especially appropriate for RRBS is prompted by the thought that researchers engaged in a WGBS study may have slightly different aims, and this goes back to your comment about hypotheses. In particular, it may be important in a WGBS analysis to discover DMRs in a *de novo* fashion, pooling results for consecutive genomic loci without the use of gene annotation. We have added remarks in the Introduction and the Discussion to clarify our aims and to briefly indicate possible extensions. There is no problem with computational resources – our pipeline is reasonably efficient.

In this revision, we have relaxed the filtering threshold to 8 instead of 10 reads per CpG, although it makes no material difference to our analysis. While the filtering does indeed remove 85% of the CpGs, it keeps more than half the counts. The number of CpGs retained in the analysis is plenty for our purposes.

In this revision, we form the MDS plot in a slightly better way and P8_6 is no longer an outlier.

In Figure 2, the meaning of “average abundance” is explained by the axis label as log count per million, i.e., coverage for that CpG divided by sequencing depth. In effect, it is the coverage for each CpG relative to other CpGs. It is independent of sequencing depth.

# Peer review: Referee response for version 1

**DOI:** 10.5256/f1000research.14317.r28484

**Published:** 2017-12-18

**Authors:** James W. MacDonald

**Affiliations:** 1 Department of Environmental and Occupational Health Sciences, University of Washington, Seattle, WA, USA

This is primarily a software pipeline article, showing how to use the Bioconductor edgeR package to analyze RRBS data, but to a certain extent is also a methods paper, as to my knowledge this is the first proposal for directly analyzing count data rather than converting to either ratios and using a beta-binomial, or to logits and using conventional linear modeling. This is an interesting idea, and should be explored further, but for this manuscript the main goal is to present the software pipeline.

 The authors progress through each step of the pipeline, clearly describing each step as well as providing code (and links to the underlying data), so readers can easily understand the process and get some hands on experience as well.

 The code is clearly written, and as straightforward as one could expect for a relatively complex analysis. However, I would prefer to see more consistent integration with other Bioconductor packages. In particular, when reading in the raw data, the authors use a clever trick to account for the fact that not all samples have reads for the same genomic positions. This step could just as easily be accomplished using the Bioconductor GenomicRanges package, which is intended for manipulating genomic data. In fact, the authors use GenomicRanges later in the pipeline to subset the methylation data to just gene promoter sites, so it would be more natural to start with a GRanges if you will need one later anyway.

 Otherwise this is a good article that clearly shows how one could use an innovative method to analyze RRBS data using the edgeR package.

 Typos: 

 Under a small example section, (BvsA_MvsU) estimates the difference in logit proportions of **mythylated**

I have read this submission. I believe that I have an appropriate level of expertise to confirm that it is of an acceptable scientific standard.

# Response

**Published:** 2018-10-01

**Authors:** Gordon Smyth

**Affiliations:** Walter and Eliza Hall Institute of Medical Research, Australia

Thank you for your positive report on our article. You’re right, there is a novel methods proposal in our article, which we will seek to justify in more detail in a methodological article elsewhere, but the current article concentrates on the software pipeline.

In this revision, we have introduced new functions readBismark2DGE() and nearestTSS() to simplify the workflow. These functions allow us to eliminate from the workflow any code chunks that involve substantial programming, making the workflow considerably easier for users. We felt the original code chunk to find CpGs in promoters in particular was too complex for the purposes of our article and assumed too much user knowledge.

I have used GenomicRanges for other projects ( https://f1000research.com/articles/4-1080 ) but it doesn’t help with the current workflow. The file reading code would be similar in length and speed with or without GenomicRanges, so we tend to give preference to R base. In any case, we have eliminated that code entirely, as explained in our discussion with Peter Hickey.

The new nearestTSS() function is more informative than the old code, as well as being fast. It has the added advantage of using the Bioconductor organism package, which has more up-to-date TSS annotation than the transcript package.

# Peer review: Referee response for version 1

**DOI:** 10.5256/f1000research.14317.r28483

**Published:** 2017-12-07

**Authors:** Simon Andrews

**Affiliations:** 1 Bioinformatics Group, Babraham Institute, Cambridge, UK

Chen *et al* present an interesting re-application of the EdgeR analysis package to the analysis of bisulphite sequencing data.  The method they propose would utilise the existing negative biomial models within EdgeR and would potentially provide the power which comes with the linear model framework to bisulphite data.  The method described requires no changes to EdgeR itself, and merely describes a suitable formulation of design matrix to allow this to be applied to bisulphite data.

 The article is generally well written and the authors go to great lengths to break down and describe the method.  They also provide a page from which all of the underlying data and code can be obtained and I was able to reproduce the results, and independently verify them in a parallel analysis.

 The main thing which I struggled with was some of the detail in the description of the method itself.  There were some parts which I wasn't clear on, and some nomenclature which didn't help in understanding the explanation.  I'll try to lay out my concerns below:

 1) In the small example I completely understand that the authors wanted to keep this as simple as possible, but it might have helped to have had 2 samples per condition so that the full complexity of the method is visible. 

 2) There is a typo in the code for the small example so it doesn't run as is.  The list function on line 2 has an extra bracket at the end.

 3) The nomenclature in the small example is inconsistent.  You have samples 1 and 2, but (in the table) also conditions 1 and 2, but in the code the conditions are A and B.  If you had Samples 1,2,3,4 in conditions A and B this might help to alleviate some of the confusion.

 4) In the small example description you say that A_MvsU estimates the log ratio for Sample1, but it wasn't clear to me why this would apply to only Sample 1 since the factor has a 1 against the meth count for both samples 1 and 2

 In the expanded examples there were also some points on which I wasn't clear.

 5) You calculate a single dispersion parameter for all data points and say that in contrast to RNA-Seq there is no global trend to follow.  It wasn't clear to be exactly why this is since read count and methlyation level would all affect the dispersion - is it simply because these factors are explicitly accounted for in the linear model?

 6) In the design matrix for the RRBS it wasn't clear why the first column was all 1s, whereas the rest obviously matched the condition from which they came.  This also contrasted with the simple example where the structure wasn't like this. Is this because you were comparing both P7 and P8 to P6?

 7) I think this is possibly the same thing as point 4, but you say that the Me column represents the methylation level in P6, but again this highlights the methylated values in all samples, so why only P6?

 For the final results obtained it would have been nice to show the general level of concordance with running the same analysis through one of the beta-distribution models to either show general agreement, or to generally explain any major differences.

 Minor points:

 In the introduction you say that "40% of mammalian genes and 70% of human genes have CpG islands enriched in their promoter regions".  Enriched probably isn't the right word to use (or you need to say that CpGs are enriched rather than islands).  The difference between 'humans' and 'mammals' is also somewhat contentious - non-human mammals certainly have weaker CpG islands which get missed by CpG island prediction tools, but for example in mouse Illingworth et al showed that if you use CpG binding protein ChIP that you can see about the same number of islands in both species.

 It's also not really fair to say that CpG methylation in promoters is "generally" associated with repression of transcription.  There is a categorical expression level shift associated with the presence/absense of CpG islands, but you can make a Dnmt1 knockout which removes pretty much all methlyation from the genome and for the vast majority of genes their transcription is completely unaffected.

 P3 "with large genome" should be "with large genomes"

 P5 "mythylated" should be "methylated"

I have read this submission. I believe that I have an appropriate level of expertise to confirm that it is of an acceptable scientific standard, however I have significant reservations, as outlined above.

# Response

**Published:** 2018-09-30

**Authors:** Gordon Smyth

**Affiliations:** Walter and Eliza Hall Institute of Medical Research, Australia

Thank you for your thoughtful comments on our article.

 We have rewritten the Introduction to take account of your comments and have fixed all typos. We have also expanded the expository section on the NB linear modeling approach to five pages instead of one.

 The purpose of the first toy example (Table 1) is to introduce the key mathematical idea that underlies our article, which is the use of linear model terms to account for read coverage at each CpG, and to relate this to the more familiar beta-binomial modeling approach. This idea is already manifest in the very small dataset without replicates, making the one-sample example the most fundamental and effective context in which to introduce the idea. We agree however that the condition labels in Table 1 should be A and B.

 Following your remarks, we have included a second example with 2 samples per condition (Table 2). Keep in mind though that the edgeR approach to methylation however is very powerful and flexible and its full complexity is still far from being visible in a single locus example like this. To see more of the features in play, one has to work through the full data example. We have however taken the opportunity to preview more ideas in the expanded linear modeling introduction, including dispersion estimation and the use of contrasts.

 You asked about the A_MvsU coefficient computation. This is a standard linear modeling result in R but one that does confuse a lot of people when they see it for the first time. You will see analogous results explained in the limma and edgeR User’s Guides. Anyway, we have rewritten the first example in a different way so that the coefficients are defined more explicitly and their calculation is explained in full arithmetic detail.

 The original implicit formulation of the logFC is now presented in the section “Another way to make the design matrix”. Consider the last two columns of the design matrix in that section, which are now called “Intercept” and “ConditionB”, and write β _A_ and β _BvsA_ for the corresponding two coefficients. The interpretation of these coefficients becomes clear by multiplying the coefficients by the design matrix. For samples corresponding to condition A, the methylation level is represented by β _A_ alone because the last column of the design matrix is zero (rows 1 and 3). When condition B is applied, the methylation is modelled by β _A_ + β _BvsA_ (rows 5 and 7). So it is apparent that β _A_ must represent the methylation status for condition A while β _BvsA0_ must be the difference in methylation between B and A. Users don’t need to follow this mathematics, we just present it here for completeness.

 You also asked about the first column of the design matrix. It doesn’t matter whether the first column is all 1s or not, as long as the leading columns of the design matrix span the sample effects. We have added a small example on pages 3-4 to demonstrate this. The first few columns of the design matrix model the sample coverages, and these columns are quite independent of the remaining columns which model the treatment conditions. We can compare the treatment conditions in any way we wish regardless of how the design matrix is parametrized. We have added some material to the introduction to try to explain this but, in any case, it isn’t something that a user needs to worry about.

 In this revision, we have introduced a new function modelMatrixMeth to create the design matrix automatically, so users now only need to focus on the treatment conditions, not on how to adjust for the sample effects.

 There is no reason why the dispersion should be a function of read count or of methylation level. The purpose of the NB statistical model is to capture the technical mean-variance trends so that the dispersion parameter can be interpreted independently of these things. We have added a sub-section on dispersion estimation to page 7 to try to make this clear.

 Regarding concordance with other methylation analysis software, we do not know of any beta-binomial modeling software that is able to conduct an equivalent analysis to that presented in our article. We have formed a contrast between luminal cells and the two basal populations, and no other software can do that as far as we know. This is an example of the extra flexibility provided by our approach. The DSS Bioconductor package is documented to be able to form general contrasts between treatment conditions, but this is not effective in practice because it does not distinguish hyper from hypo methylation for a contrast.

 Our experience is that edgeR gives better DM results than beta-binomial software but it would be unfair for us to claim that in our article without undertaking a full comparison study with rigorous benchmarking. Our plan is to publish such a comparison elsewhere.
